# Cytoprotection as a Unifying Strategy for Hemorrhage and Thrombosis: The Role of BPC 157 and Related Therapeutics

**DOI:** 10.3390/ph19030463

**Published:** 2026-03-12

**Authors:** Predrag Sikiric, Ivan Barisic, Mario Udovicic, Martina Lovric Bencic, Diana Balenovic, Dean Strinic, Gordana Zivanovic Posilovic, Sandra Uzun, Hrvoje Vranes, Ivan Krezic, Marin Lozic, Vasilije Stambolija, Ivica Premuzic Mestrovic, Lidija Beketic Oreskovic, Luka Kalogjera, Sanja Strbe, Suncana Sikiric, Laura Tomic, Mirjana Stupnisek, Mario Kordic, Ante Tvrdeic, Sven Seiwerth, Alenka Boban Blagaic, Anita Skrtic

**Affiliations:** 1 Department of Pharmacology, School of Medicine, University of Zagreb, 10000 Zagreb, Croatiamarioudovicic@gmail.com (M.U.);; 2 Department of Anesthesiology, Resuscitation and Intensive Care, University Hospital Centre Zagreb, 10000 Zagreb, Croatia; 3 Department of Pediatric and Preventive Dentistry, School of Dental Medicine, University of Zagreb, 10000 Zagreb, Croatia; 4 Department of Pathology, School of Medicine, University of Zagreb, 10000 Zagreb, Croatia

**Keywords:** cytoprotection, hemorrhage, thrombosis, BPC 157, vascular homeostasis, wound healing, arrhythmias, Virchow triad

## Abstract

This review presents an innovative and timely exploration of how cytoprotection can serve as a cohesive therapeutic approach by which to address the hemorrhage–thrombosis paradox. Presenting counteraction of both hemorrhage and thrombosis as phase-dependent outcomes of vascular dysregulation, the manuscript synthesizes conceptual, experimental, and clinical evidence into a unified systems-level model focused on the stable gastric pentadecapeptide BPC 157, which acts as a cytoprotective mediator. In rodents, BPC 157 can simultaneously counteract hemorrhage and thrombosis without directly affecting the coagulation cascade (aggregometry, thromboelastometry). This cytoprotective framework (decreased hemorrhage, decreased thrombosis) stands with presentation of both hemorrhage and thrombosis in the wound, arrhythmias, and Virchow triad, and resolution of these disturbances. As proof of the concept (full cytoprotective effect), a vasoprotective cytoprotective mediator capable of bidirectional regulation, BPC 157, is effective for wound healing, arrhythmia control, and normalization of Virchow’s triad (i.e., following major injuries, occlusion/occlusion-like syndromes). As a comparison from a cytoprotective (partial vs. full) standpoint, conventional agents—anticoagulants, antiplatelet drugs, and fibrinolytics—provide only partial protection by targeting isolated components of hemostasis. Beta blockers, calcium channel blockers, prostaglandins, NO modulators, ACE inhibitors, and statins each exert broader cytoprotective effects; however, these actions remain incomplete and context-dependent, typically unidirectional, dose-limited, or are achieved at the expense of opposing pathological risks. Contrarily, for BPC 157, decreased hemorrhage (including both anticoagulants and antiplatelet agents), decreased thrombosis, effective wound healing, arrhythmia control, and normalization of Virchow’s triad involve preservation of endothelial integrity, normalization of microcirculation, modulation of the NO system, stabilization of hemostatic balance, and recruitment of adaptive collateral pathways. Nevertheless, reliance on preclinical models necessitates further clinical validation.

## 1. Introduction

This review attempts to present an innovative and timely exploration of how cytoprotection [1,2,3,4,5,6,7,8,9] can serve as a cohesive therapeutic approach to tackle the hemorrhage–thrombosis paradox [10,11,12,13,14,15]. Presenting counteraction of both hemorrhage and thrombosis as phase-dependent outcomes of vascular dysregulation, the manuscript synthesizes conceptual, experimental, and clinical evidence into a unified systems-level model focused on the stable gastric pentadecapeptide BPC 157, which acts as a cytoprotective mediator [16,17,18,19,20,21,22,23,24,25,26]. In rodents, BPC 157 can simultaneously counteract hemorrhage and thrombosis without directly affecting the coagulation cascade (aggregometry, thromboelastometry) [16,17,18,19,20,21,22,23,24,25,26].

We advocate that the cytoprotection concept, as a process to preserve and reestablish homeostasis [1,2,3,4,5,6,7,8,9], challenges the classical view of hemostasis and thrombosis as opposing pathological states [10,11,12]. Within this conventional framework, anticoagulants prevent thrombosis but induce bleeding, whereas procoagulants control bleeding while promoting thrombosis [13]. In contrast, the cytoprotection concept of vascular integrity frames both hemostasis and thrombosis as processes with an essential dual capacity to preserve tissue integrity and restore homeostasis, irrespective of the nature of the injury. Accordingly, in bleeding/thrombosis disorders, true cytoprotection is the bidirectional normalization of vascular and cellular homeostasis, counteracting both hemorrhage and thrombosis without inducing opposite-pathology toxicity. Importantly, as a universal reversal strategy, this approach would transcend the heterogeneity of anticoagulants and the involvement of distinct coagulation pathways. At present, unlike specific antagonists, no single agent is applicable to counteract both heparin- and warfarin-induced bleeding [14,15], a common antidote is lacking.

Translating this concept into practice through the application of a cytoprotective agent (i.e., the stable gastric pentadecapeptide BPC 157 [16,17,18,19,20,21,22,23,24,25,26]) may be important. This implies that a true cytoprotective therapy should normalize pathological bleeding or thrombosis in either direction without provoking opposing toxicity [16,17,18,19,20,21,22,23,24,25,26]. Such coordinated and balanced control of bleeding and thrombosis raises the longstanding question of an “ideal agent” capable of preserving therapeutic efficacy while avoiding adverse effects.

From a cytoprotective viewpoint—where agents may share partial cytoprotective properties (e.g., antioxidative, gastroprotective, cardioprotective, or organ-protective effects)—partial solutions have been achieved with conventional anticoagulants, antiplatelet agents, and fibrinolytics [10,11,12,13,14,15]. Ultimately, however, given that the cytoprotection concept (cell protection) was born in the stomach [1,2,3,4,5,6,7,8,9], the stable gastric pentadecapeptide BPC 157 has emerged as a particularly relevant cytoprotective therapy [16,17,18,19,20,21,22,23,24,25,26]. This study also includes other agents exhibiting cytoprotective properties, including prostaglandin analogues [1,2,3,4,5,6,7,8,9], antioxidants [27,28,29], angiotensin-converting enzyme inhibitors [30], statins [31,32], beta blockers [33,34], and calcium channel blockers [35,36]. These illustrate that cytoprotection is not a distinct pharmacological class but rather a shared therapeutic property across diverse drug groups. Peptides such as PDGF-BB [37], TGF-β1 [38], FGF-2/bFGF [39], VEGF [40], IGF-1 [41], and BMPs [42] were not incorporated into this framework. Thus, cytoprotection currently represents a conceptual therapeutic effect rather than a standardized clinical drug category.

### Stable Gastric Pentadecapeptide BPC 157

Among cytoprotective agents, BPC 157 has accumulated the most extensive preclinical evidence [16,17,18,19,20,21,22,23,24,25,26]. Unlike other compounds, it is native and stable in human gastric juice for more than 24 h and has been proposed as a mediator of cytoprotection capable of transmitting gastrointestinal mucosal integrity to systemic organ protection [16,17,18,19,20,21,22,23,24,25,26]. This distinctive property underlies its pleiotropic beneficial effects. This includes efficacy via multiple routes, including oral administration. These effects are attributed to its modulatory interactions with multiple molecular pathways [43,44,45,46,47,48,49,50,51,52,53,54], particularly the nitric oxide (NO) system [16,55,56,57]. Toxicological studies have demonstrated an exceptionally favorable safety profile, with no lethal dose achieved (LD_1_ not reached) even at doses of 2 g/kg i.v. or i.g. in mice [16,17,18,19,20,21,22,23,24,25,26]. Human data remain limited but encouraging. BPC 157 was found to be effective and well tolerated in phase II clinical trials in ulcerative colitis and subsequently in small clinical studies addressing knee pain and interstitial cystitis, all without reported adverse effects [58,59,60,61]. These findings are supported by toxicological evaluations [62,63] and patient reports [64,65,66], consistent with previous assessments [16].

In hemorrhage- and thrombosis-focused experimental studies, bleeding and thrombosis were treated as specific cytoprotection targets [16,17,18,19,20,21,22,23,24,25,26], incorporating decreased hemorrhage and decreased thrombosis as part of a cytoprotection framework. The first direct evidence of BPC 157’s antithrombotic action was demonstrated in a rat abdominal aorta anastomosis model, where it counteracted thrombosis formation and reversed established clots [67]. Its capacity to normalize prolonged bleeding was subsequently shown in models involving heparin, warfarin, aspirin, and traumatic injury [68,69]. Aggregometry and thromboelastometry studies further indicated that BPC 157 restores platelet function without affecting coagulation pathways [70].

Additional evidence (decreased hemorrhage, decreased thrombosis) derives from severe models characterized by the coexistence of organ hemorrhage and widespread thrombosis, including thrombohemorrhagic disorders, major vessel occlusion, occlusion-like syndromes, and multiorgan failure [71,72,73,74,75,76,77]. These conditions were found to be induced by vascular occlusion, noxious procedures [78,79,80,81], or toxic agents [82,83,84,85,86,87], and were consistently reversed by BPC 157 therapy. A distinctive rescue mechanism involves the rapid activation of collateral circulation, particularly via the azygos vein, leading to restoration of systemic hemodynamics. Importantly, BPC 157 exhibits a modulatory interaction with the NO system, counteracting both NO inhibition and NO overactivity [16], and consistently antagonizes the adverse vascular effects of non-steroidal anti-inflammatory drugs [88].

## 2. Cytoprotection, Anticoagulants, Antiplatelet Agents, and Fibrinolytics

### 2.1. Anticoagulants and Cytoprotection: Direct Oral Anticoagulants (DOACs), Unfractionated Heparin, and Low-Molecular-Weight Heparins (LMWHs)

Many anticoagulants exhibit cytoprotective or pleiotropic effects beyond anticoagulation, including endothelial stabilization, anti-inflammatory activity, and modulation of oxidative, fibrotic, and cellular injury pathways [89,90,91,92,93,94]. Although not always described as “cytoprotection,” these actions align with the concept of preserving cellular integrity [1,2,3,4,5,6,7,8,9].

DOACs (dabigatran, rivaroxaban, apixaban, edoxaban) confer endothelial protection, reduce inflammation, prevent oxidative damage, limit fibrosis, and preserve tissue integrity via PAR signaling pathways [89,90,91]. Clinical evidence suggests that oral anticoagulants can lower inflammatory biomarkers, consistent with non-hemostatic cytoprotective effects [90]. DOACs preserve tight junction proteins and reduce endothelial permeability in vitro, further supporting their protective role [91].

LMWHs (e.g., enoxaparin) inhibit complement activation and neutrophil elastase, protecting cells in extracorporeal circulation models [92]. Factor Xa inhibition modulates PAR signaling on endothelial and immune cells, mitigating inflammation [93]. Heparin additionally protects the endothelial glycocalyx and mediates anti-inflammatory interactions independently of antithrombin, contributing to vascular protection [94].

### 2.2. Warfarin and Cytoprotection

Warfarin, in contrast, lacks cytoprotective effects and may promote vascular dysfunction [95,96,97,98,99,100,101]. By inhibiting vitamin K-dependent matrix Gla protein, warfarin accelerates arterial and valvular calcification, increases susceptibility to oxidative stress, and impairs endothelial survival [95,96,97,98,99]. Consequently, warfarin therapy reduces the vasculature’s intrinsic cytoprotective potential [100,101].

### 2.3. Antiplatelet Agents and Cytoprotection

Aspirin and P2Y12 inhibitors (clopidogrel, ticagrelor) provide antithrombotic effects along with endothelial protection, anti-inflammatory activity, and reduction of oxidative stress [102,103,104,105,106,107]. Aspirin plays a mechanistic role in foundational gastric cytoprotection studies, demonstrating prostaglandin-mediated adaptive defense via cyclooxygenase inhibition [1,3].

### 2.4. Fibrinolytics and Indirect Cytoprotection

Fibrinolytic agents (tPA, streptokinase, urokinase) primarily dissolve thrombi but can indirectly protect tissues by restoring perfusion and limiting ischemic injury, apoptosis, oxidative stress, and secondary inflammation [108,109,110,111,112]. In myocardial and cerebral ischemia, fibrinolysis reduces infarct size and tissue damage, although cytoprotection is secondary to thrombus removal [112].

### 2.5. Hemorrhage and Thrombosis: Anticoagulant Cytoprotection

Despite their preventive intent, anticoagulants can paradoxically induce thrombosis under certain conditions. Examples include heparin-induced thrombocytopenia (HIT), where antibodies against the heparin–PF4 complex cause massive platelet activation [113], and warfarin-induced skin necrosis from transient hypercoagulability due to protein C/S depletion [114,115]. DOACs may trigger thrombosis in cases of underdosing (e.g., renal impairment), and abrupt anticoagulant withdrawal can produce rebound hypercoagulability [116].

Even with low-risk alternatives (LMWHs, fondaparinux, heparinoids), residual HIT risk persists [117,118,119]. Warfarin initiation often requires bridging with UFH or LMWH to mitigate early hypercoagulability [120,121]. Emergency reversal agents (protamine for heparin and idarucizumab for dabigatran), which lack intrinsic prothrombotic properties, rapidly restore hemostasis but may be associated with thrombotic events [122,123,124,125,126]. These events are most plausibly attributable to abrupt neutralization of anticoagulation and re-exposure of the patient’s underlying prothrombotic state rather than to the direct procoagulant effects of the reversal agents.

### 2.6. Hemorrhage and Thrombosis: Antiplatelet Cytoprotection

Antiplatelet agents, though designed to prevent thrombosis, can also cause rare paradoxical events, including thrombocytopenia-induced thrombosis (e.g., GPIIb/IIIa inhibitors, thienopyridines) [127,128,129] and rebound platelet hyperreactivity after abrupt cessation, especially around percutaneous coronary interventions [130]. Temporary discontinuation of low-dose aspirin for surgery may increase cardiovascular morbidity and mortality [131].

### 2.7. Hemorrhage and Thrombosis: Fibrinolytic Cytoprotection

Fibrinolytics can paradoxically induce thrombotic events despite their clot-dissolving activity. Rapid clot lysis exposes procoagulant surfaces, activates platelets, thrombin, and coagulation factors, and may precipitate reocclusion or recurrent thrombosis, termed the “thrombolytic paradox” [132,133,134,135,136]. Plasmin generated during fibrinolysis can additionally activate platelets or endothelial cells under inflammatory conditions, temporarily increasing thrombotic risk [136].

These points are summarized in Table 1.

### 2.8. Thrombohemorrhagic Disorders: A Hemostatic Paradox of Simultaneous Thrombosis and Hemorrhage

Thrombohemorrhagic disorders, in which thrombosis and bleeding occur concurrently, reflect profound dysregulation of vascular and cellular homeostasis rather than simple deficiencies or excesses of clotting factors. Conceptually, this represents a failure of cytoprotective mechanisms that normally preserve endothelial integrity and maintain hemostatic balance. Conventional therapies have not systematically leveraged cytoprotection to address these syndromes.

Disseminated intravascular coagulation (DIC) exemplifies this paradox: uncontrolled thrombin generation causes microvascular thrombosis, while consumption of platelets and clotting factors produces bleeding. Triggers include sepsis, trauma, malignancy, and obstetric complications, involving excessive procoagulant activation, impaired anticoagulant pathways, endothelial injury, and variable fibrinolysis. Therapy focuses on treating the underlying cause, which is most effective in restoring hemostasis [137]. Supportive interventions—platelets, fresh frozen plasma, cryoprecipitate—address critical deficiencies but do not prevent ongoing microthrombosis. Low-dose heparin may be considered when thrombosis predominates, but bleeding risk must be carefully weighed [138].

Thrombotic microangiopathies (TMAs), such as thrombotic thrombocytopenic purpura (TTP) and atypical hemolytic uremic syndrome (aHUS), similarly demonstrate this dual risk. Widespread microvascular platelet-rich thrombi cause thrombocytopenia, hemolytic anemia, and organ ischemia while predisposing to bleeding. Plasma exchange in TTP removes ultra-large von Willebrand factor multimers and pathogenic autoantibodies, reducing thrombosis and mortality [139]. Complement inhibition with eculizumab in aHUS prevents microthrombus formation and preserves hematologic and renal function [140,141]. Platelet transfusions are generally reserved for life-threatening hemorrhage.

Effective management requires early recognition and mechanism-directed therapy, as supportive measures alone are insufficient. Interventions—anticoagulation, plasma exchange, complement inhibition—must be tailored to dominant pathophysiology, continuously balancing thrombosis and hemorrhage, guided by close laboratory monitoring.

These syndromes illustrate a fundamental limitation of conventional anticoagulants, antiplatelets, and fibrinolytics: by acting on discrete hemostatic components, they inevitably exchange thrombotic risk for bleeding risk or vice versa. This paradox highlights the absence of a true cytoprotective mechanism capable of preserving endothelial integrity, maintaining cellular homeostasis, and restoring physiologic hemostasis.

Agents such as BPC 157, with demonstrated pleiotropic cytoprotective effects, may offer a therapeutic approach that directly addresses this hemostatic paradox by simultaneously supporting vascular and cellular homeostasis while counteracting both thrombosis and hemorrhage.

### 2.9. BPC 157 Cytoprotection

Conceptual theory implementation (decreased hemorrhage, decreased thrombosis) holds the cytoprotection concept [1,2,3,4,5,6,7,8,9], though it is not organ specific (although formed in the stomach). A systemic protection (cytoprotection → organoprotection, i.e., gastrointestinal tract maintenance transmitted to other organs therapy) is achieved by administering cytoprotective agents and via the innate pleiotropic beneficial effects attributed to those agents [1,2,3,4,5,6,7,8,9]. This principle is much more attributable to BPC 157 than to other cytoprotection agents mentioned before [16,17,18,19,20,21,22,23,24,25,26].

There, the stable gastric pentadecapeptide BPC 157, holds particular cardioprotection, anti-thrombotic, anti-hemorrhagic, anti-arrhythmic, and vascular recovery potential, particularly during ischemia/reperfusion injury recovery [16,17,18,19,20,21,22,23,24,25,26]. Specifically, principle verification (decreased hemorrhage, decreased thrombosis) includes a complex experimental model range [16,17,18,19,20,21,22,23,24,25,26] that encompasses vessel anastomosis [67], tail or leg amputation, anticoagulants, antiplatelets [68,69], organ perforation [81,142], vessel occlusion [71,72,73,74,75,76], severe injury induction [78,79,80,81], agent application [82,83,84,85,86,87], and occlusion/occlusion-like syndrome. Also included are counteractions of those aggravated by NO/NO synthase (NOS) blockade (L-NAME) or NO overactivity (L-arginine) [69,85]. Notably, as pointed out, this range also includes the simultaneous presentation of organ hemorrhage and thrombosis and counteraction. Additional evidence that BPC 157 may rescue thrombocyte function without affecting coagulation pathways [68,69] includes aggregometry and thromboelastometry studies [70]. Notably, BPC 157 may recover prostaglandin function, given the counteraction of NSAID-induced adverse effects [88].

### 2.10. Verification of the Cytoprotective Effects (Decreased Hemorrhage, Decreased Thrombosis) Through Three Confirmatory Principles: (i) Wound, (ii) Arrhythmias, and (iii) Virchow Triad

**Figure 1:**
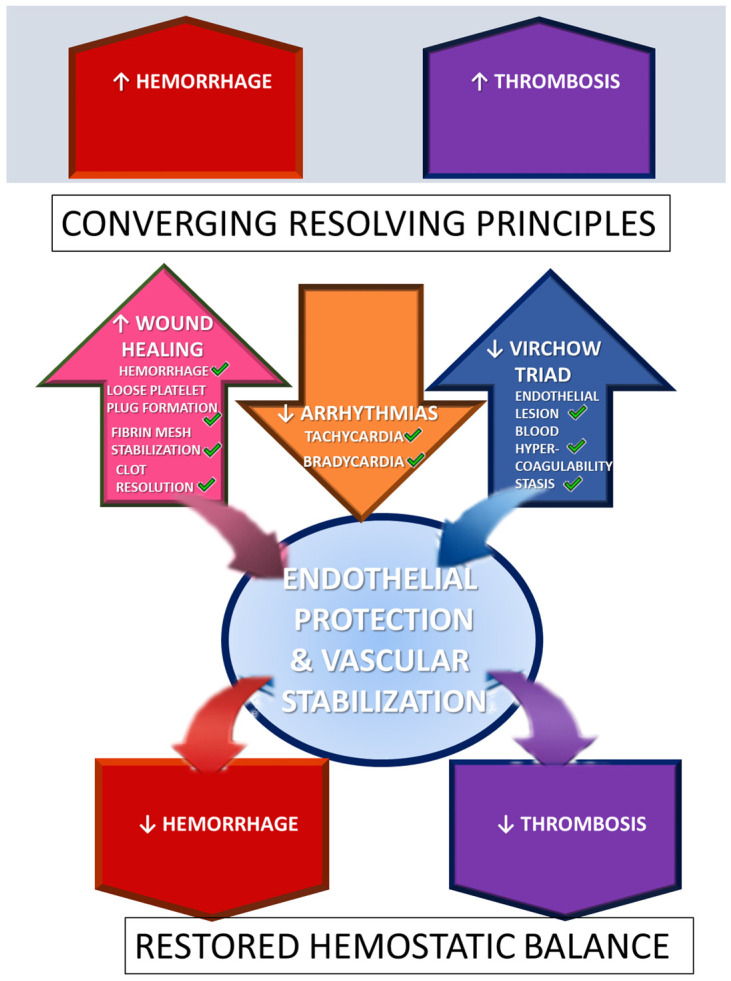
Converging resolving principles. Considering that hemorrhage and thrombosis are both involved in the wound, arrhythmia, and Virchow triad, the effects on the circumstances of wound healing, arrhythmia, and Virchow triad were considered as key proof of the agent’s counteracting activity (green tick marks) on either hemorrhage or thrombosis, or counteraction of both hemorrhage and thrombosis [16,17,18,19,20,21,22,23,24,25,26,145].

These cytoprotective effects, decreased hemorrhage and decreased thrombosis, conceptually anchor three confirmatory principles that provide presentation of both hemorrhage and thrombosis—(i) wound, (ii) arrhythmias, and (iii) Virchow triad. These (decreased hemorrhage, decreased thrombosis) have to be reversed in order to achieve (i) wound healing, (ii) arrhythmia mitigation, and (iii) normalization of Virchow’s triad (see Figure 1). BPC 157 therapy full accomplishes all of these points [16,17,18,19,20,21,22,23,24,25,26], which is significant given that both hemorrhage and thrombosis regularly need to be counteracted simultaneously.

Wound healing, in particular, encompasses all four major hemostatic events—vascular constriction, loose platelet plug formation, fibrin mesh stabilization, and clot resolution—which occur in a coordinated sequence after vascular injury [143,144,145]. Accordingly, in hemorrhage/thrombosis terms, the principle ↑ wound healing = ↓ hemorrhage and ↓ thrombosis applies, as agents that promote wound healing, such as BPC 157, effectively reduce both bleeding and thrombotic risk. Taken as a conceptual model, these confirmatory endpoints also provide a framework for evaluating other cytoprotective agents, although none matches the comprehensive systemic efficacy of BPC 157.

## 3. Wound Healing

### 3.1. BPC 157: Wound Healing as a Resolving Principle for Hemorrhage/Thrombosis Therapy

Wound healing has long been recognized as an inherent component of the cytoprotection concept and a central mediator of cytoprotective agent activity [1,2,3,4,5,6,7,8,9,16,17,18,19,20,21,22,23,24,25,26]. In the context of BPC 157 therapy, wound healing provides a unifying framework for resolving the apparent paradox of simultaneously counteracting hemorrhage and thrombosis [145]. This can be particularly seen given the comparative studies reviewed [145]. As a conceptual principle, we have proposed that any agent truly promoting wound healing must, by necessity, normalize both bleeding and thrombosis, depending on the phase of vascular repair [68].

Early BPC 157 studies demonstrated endothelium maintenance. Initially, this was in the context of alcohol-induced gastric lesions [146], and later in models of thrombosis and prolonged bleeding, including rat abdominal aorta anastomosis [67], anticoagulant (heparin, warfarin) and antiplatelet exposure [68,69], limb amputation, organ perforation [81,142], vessel occlusion [71,72,73,74,75,76,77], severe injury induction [78,79,80,81], and agents’ application [82,83,84,85,86,87] in occlusion/occlusion-like syndromes [71,72,73,74,75,76,77,78,79,80,81,82,83,84,85,86,87]. These models also included NO/NOS dysregulation, with L-NAME or L-arginine, and conditions where hemorrhage and thrombosis occurred simultaneously [69,85]. BPC 157 restored thrombocyte function without affecting coagulation pathways [70] and counteracted NSAID-induced prostaglandin dysfunction [88].

The dual therapeutic effect of BPC 157, context-dependent, aligns with the four sequential phases of physiological wound healing: (i) vascular constriction, (ii) loose platelet plug formation, (iii) fibrin mesh stabilization, and (iv) clot resolution with restoration of vascular patency [143,144,145,147]. Effective wound healing, therefore, inherently supports early hemostasis, thrombus formation, and timely clot dissolution. Failure in any phase can result in persistent hemorrhage or pathological thrombosis, highlighting that bleeding and thrombosis are not opposing events but temporally and functionally linked stages of the repair process [145]. This principle establishes the therapeutic design requirement for phase-sensitive, cytoprotective interventions with robust wound healing capacity as the primary endpoint.

Experimental evidence (decreased hemorrhage, decreased thrombosis) confirms BPC 157’s potent wound healing activity across multiple tissues and injury types. These include skin wounds, deep burns [53,54,63,148,149,150,151,152], tendons [50,51,153,154,155,156,157,158], ligaments [159], muscle injuries [47,158,160,161,162,163], osteotendinous and myotendinous junctions [153,154,157,164], and muscle-to-bone attachments [165]. Corneal ulcer healing, including restoration of transparency and counteraction of neovascularization, further exemplifies its multi-tissue efficacy [21,22,23,26,166]. Internal wound repair is also robust, as demonstrated by recovery of anastomoses [167] and fistulas [168], both external [169,170,171,172,173] and internal [174,175,176,177]. These outcomes confirm early hemostasis, subsequent clot removal, and microvascular restoration.

Overall, BPC 157’s systemic wound healing capacity substantiates its dual role in decreasing hemorrhage and thrombosis and provides a conceptual and experimental framework for evaluating other cytoprotective agents, though none match its comprehensive efficacy.

Notably, as part of the increasing interest for soft tissue injuries healing and BPC 157 efficacy, several reviews have recently appeared [178,179,180,181,182,183,184,185,186,187,188,189,190].

### 3.2. Cytoprotective Effects of Prostaglandins, Statins, ACE Inhibitors, Beta Blockers, Ca Channel Blockers, and NO Modulators as Possibilities for Wound Healing

When examined from the perspective of other pharmacological agents that exhibit partial cytoprotective or pleiotropic effects, supportive evidence for wound healing is present but notably inconsistent. Unlike BPC 157, these agents were not developed as cytoprotective therapies and do not uniformly engage the coordinated, multistage repair program required for effective wound healing.

Prostaglandins, which historically represent the first mediators of the cytoprotection concept, demonstrate a biphasic influence on wound repair [191]. Under physiological conditions, prostaglandins generally promote healing through vasodilation, angiogenesis, and epithelial restitution [192,193,194]. However, excessive prostaglandin signaling—particularly elevated PGE_2_—may exacerbate inflammation, fibrosis, and scarring, resulting in impaired or dysregulated healing [195].

Statins also exhibit dose- and context-dependent effects. At low-to-moderate doses, statins may enhance wound healing via anti-inflammatory actions, endothelial stabilization, and improved nitric oxide bioavailability. In contrast, higher doses have been associated with delayed tissue repair, likely due to excessive inhibition of cellular proliferation and prenylation pathways essential for regeneration [196].

ACE inhibitors generally support wound healing, presumably through improved microcirculation, bradykinin-mediated vasodilation, and modulation of inflammatory responses [197,198,199].

Beta blockers exhibit complex effects: while some studies report accelerated wound closure—possibly through reduced stress signaling and catecholamine effects—others report delayed healing, reflecting interference with perfusion, angiogenesis, or keratinocyte migration [200,201,202,203,204].

Calcium channel blockers are more consistently associated with improved wound healing, likely through enhanced microvascular perfusion, reduced vasospasm, and favorable effects on fibroblast and endothelial function [205,206,207,208,209,210,211].

Nitric oxide (NO) plays a central physiological role in wound repair, regulating angiogenesis, inflammation, fibroblast activity, collagen deposition, and re-epithelialization [212,213]. Accordingly, NO donors and restoration of nitric oxide synthase (NOS) activity accelerate wound closure [214,215,216], whereas NOS inhibition or NO deficiency delays healing, establishing NO modulation as a pro-healing strategy [217,218,219].

Overall, although these agents can influence selected components of wound repair, their effects are heterogeneous, dose-dependent, and mechanistically restricted, lacking the coordinated control of all of the phases of healing that are characteristic of true cytoprotection.

### 3.3. Prostaglandins, Statins, ACE Inhibitors, Beta Blockers, Ca Channel Blockers, and NO Modulators: Wound Healing as an Inconsistent Resolving Principle for Hemorrhage/Thrombosis Therapy

When the wound-healing capacity of these agents is examined in relation to the proposed cytoprotection principle linking enhanced healing with reduced hemorrhage and thrombosis (↑ wound healing = ↓ hemorrhage and ↓ thrombosis), the relationship proves inconsistent and context dependent. Unlike BPC 157, these agents do not reliably translate partial wound-healing effects into balanced hemostatic normalization.

Prostaglandins, despite their pro-healing actions, may increase bleeding risk through platelet inhibition and vasodilation [220,221,222]. Prostacyclin analogs such as iloprost are clinically used to prevent thrombosis in peripheral vascular disease and dialysis grafts, illustrating antithrombotic efficacy but at the expense of increased bleeding tendency [220].

ACE inhibitors may increase bleeding risk, particularly gastrointestinal bleeding, more frequently than angiotensin receptor blockers, consistent with bradykinin-mediated increases in vascular permeability [223,224]. Nevertheless, large cardiovascular trials demonstrate that ACE inhibitors reduce myocardial infarction and stroke incidence, indicating overall neutral or modestly antithrombotic effects [225,226,227]. Rare reports of thrombosis exist but are considered context dependent and not intrinsic pharmacological properties of this drug class [228,229].

Beta blockers generally reduce bleeding risk [229,230], yet earlier generations may reduce coronary flow at lower heart rates, potentially increasing thrombotic susceptibility [231,232]. Moreover, excessive beta blockade, as reported with sotalol overdose, may precipitate severe occlusion or occlusion-like syndromes accompanied by widespread thrombosis [84].

For calcium channel blockers, epidemiological data suggest a possible association with gastrointestinal bleeding [233], while, in patients with nonvalvular atrial fibrillation receiving oral anticoagulants and dihydropyridine, calcium channel blocker use has been associated with a higher incidence of ischemic stroke compared with non-use [234].

Statins, in contrast, are associated with reduced bleeding risk [235] and a lower incidence of venous thromboembolism in humans [236], yet these effects do not consistently align with wound-healing outcomes.

Nitric oxide represents a physiological antiplatelet and anticoagulant mediator; thus, enhancement of NO signaling, particularly at systemic or high doses, may increase bleeding risk. Conversely, NOS inhibition with L-NAME is prothrombotic, underscoring the narrow therapeutic window of NO-based modulation [237,238].

Collectively, these observations indicate that, while many pleiotropic cardiovascular agents can influence wound healing, bleeding, or thrombosis individually, they do so by shifting isolated pathways rather than restoring vascular homeostasis as an integrated process. Their inability to coordinate all four phases of wound healing—hemostasis, platelet function, fibrin stabilization, and timely clot resolution—explains why they fail to consistently resolve the hemorrhage–thrombosis paradox. This limitation sharply contrasts with the cytoprotective profile of BPC 157, which uniquely fulfills the wound-healing–hemostasis continuum as a unified therapeutic principle.

These comparative effects on wound healing, and consequently, on hemorrhage and thrombosis of prostaglandins, beta blockers, Ca channel blockers, ACE inhibitors, and NO modulators were summarized in Table 2.

Thereby, as shown in Table 2, it seems that, with all mentioned agents—prostaglandins, beta blockers, Ca channel blockers, ACE inhibitors, and NO modulators—that are supposed to have some cytoprotective capabilities, and which are thereby elaborated for wound healing/hemorrhage/thrombosis relation, the ↑wound healing = ↓ hemorrhage ↓ thrombosis remains unachieved and is not combined in a consequent chain of events.

### 3.4. Cytoprotective Effects of Anticoagulants, Antiplatelets, and Fibrinolytics as Possibilities for Wound Healing

In contrast to BPC 157, anticoagulants, antiplatelet agents, and fibrinolytics do not consistently satisfy the principle that enhanced wound healing translates into reduced hemorrhage and reduced thrombosis. Rather than coordinating the sequential phases of vascular repair, these agents act predominantly through unidirectional interference with individual hemostatic components, thereby altering wound healing in a context- and phase-dependent, but non-integrative, manner.

Heparin illustrates this limitation. While some studies demonstrate enhanced wound healing, attributed to anti-inflammatory effects, growth factor interactions, or endothelial modulation [239,240,241], others report impaired healing, delayed epithelialization, or increased wound complications [242,243,244]. These divergent outcomes reflect the absence of phase-sensitive regulation: heparin may facilitate early events but interfere with later stabilization or remodeling phases, preventing consistent restoration of vascular integrity.

Warfarin more uniformly delays wound healing [245,246,247], consistent with its inhibition of vitamin K-dependent proteins involved in tissue repair, extracellular matrix regulation, and vascular cell survival. This effect underscores a fundamental limitation of anticoagulation strategies that suppress physiological repair mechanisms rather than modulating them.

Antiplatelet agents similarly delay wound healing [248,249,250], reflecting the impaired platelet-derived signaling required for early hemostasis, growth factor release, and subsequent tissue regeneration. Fibrinolytic agents likewise delay wound healing [251,252,253], as premature or excessive clot dissolution disrupts the scaffold formation necessary for stable repair.

Collectively, these findings indicate that conventional antithrombotic agents do not support wound healing as a unified physiological process. Instead, they selectively inhibit or exaggerate specific phases, thereby failing to integrate bleeding control, clot formation, stabilization, and resolution into a coherent reparative sequence.

### 3.5. Concluding Remarks on Wound Healing–Hemorrhage–Thrombosis Relations

Wound healing represents an inherent physiological organizing principle of cytoprotection, encompassing all four essential hemostatic events—vascular constriction, loose platelet plug formation, fibrin clot stabilization, and clot dissolution—which occur in a tightly regulated temporal sequence following vascular injury. Successful healing requires that each phase is initiated, supported, and terminated appropriately; failure at any stage results in persistent hemorrhage, pathological thrombosis, or defective tissue repair.

Within this framework, hemorrhage and thrombosis are not opposing pathological states but phase-dependent manifestations of the same repair process. Accordingly, the relationship ↑ wound healing = ↓ hemorrhage and ↓ thrombosis reflects restoration of physiological homeostasis rather than pharmacological suppression of coagulation or platelet activity.

The consistent observation that BPC 157 simultaneously reduces bleeding and thrombosis therefore indicates a unique capacity to integrate wound healing with hemostatic regulation. In cytoprotection terms, this implies early hemostasis when bleeding predominates, timely clot resolution when thrombosis emerges, microvascular restoration, and normalization of platelet function—without direct interference with coagulation pathways. The outcome is a balanced, phase-sensitive modulation that follows physiological repair logic.

Other agents may influence individual components of wound healing or hemostasis, but none consistently achieves both reduced hemorrhage and reduced thrombosis in a coordinated, phase-dependent manner. Thus, BPC 157 exemplifies a cytoprotective strategy that aligns therapeutic intervention with endogenous repair mechanisms, redefining hemorrhage and thrombosis as resolvable phases of vascular healing rather than mutually exclusive targets.

### 3.6. Concluding Remarks on Wound Healing/Hemorrhage/Thrombosis Relations and BPC 157/NO System Relation

It can also be argued that such a balance would require an applicable dual effect that can be seen in both heparin and warfarin, given that they are essential as hemorrhage models [254,255,256,257,258].

Notably, unlike BPC 157 therapy studies with both heparin and warfarin [68,69], none of the mentioned agents, sharing some cytoprotection capabilities, prostaglandins, beta blockers, Ca channel blockers, and ACE inhibitors, were tested with heparin or warfarin bleeding. Likewise, they were not used in combination with targeted modulation of the NO system.

The only direct study combining NO agents with heparin and warfarin is that with regard to the effects of BPC 157 [69]. Thereby, the formula by which BPC 157 ↑ wound healing = ↓ hemorrhage and ↓ thrombosis stands with the consistent dual context-dependent effect, on both heparin and warfarin, in which BPC 157 counteracts both L-NAME pro-thrombotic effects, as well as an L-arginine anti-coagulant effect [69]. These effects occur along with the counteraction of other effects of NO agents, i.e., L-NAME-induced hypertension, and L-arginine-induced hypotension [16,54,55,56]. Notably, such counteracting effects have occurred in a large number of targets (i.e., more than 80), suggesting a general BPC 157/NO system relation [16,56]. Additionally, BPC 157 induces the NO release, resistant to L-NAME, that counteracts the NO release induced by L-arginine [16,54,55,56]. Indicating restoration of redox balance [16], BPC 157 ↑ wound healing = ↓ hemorrhage and ↓ thrombosis correlates with the findings that BPC 157 therapy normalizes NO tissue levels, either increased or decreased, along with a counteraction of the increased MDA values [16].

In conclusion, indicative therapy occurred for hemorrhage, thrombosis, hypertension, hypotension, and thrombocytes’ function (without affecting the coagulation cascade) [16,54,55,56,67,68,69,70]. In addition, BPC 157 therapy affects signaling pathways controlling vasomotor tone [45,46,47] (VEGFR2-Akt-eNOS and Src-Caveolin-1-eNOS). At the general level, this dual (modulatory) action (i.e., either of pro-coagulant effects, anti-coagulant effects, hypertension, and hypotension reversed toward normal) also applies to the NO system’s effects as a whole [16,54,55,56,67,68,69,70]. Such a role for BPC 157 therapy could be essential amid the dual role of NO, as both an inhibition and an uncontrolled excess of NO could lead to significant damage [259,260]. Consequently, in providing the essential role of NO to wound healing [261], BPC 157’s ability to restore NO system homeostasis may represent a central mechanism underlying its wide-ranging therapeutic potential, represented as the maintained equation of ↑ wound healing = ↓ hemorrhage and ↓ thrombosis [16,45,46,47,54,55,56,67,68,69,70,145].

## 4. Arrhythmias

As a conceptual principle, the ↑ wound healing = ↓ hemorrhage and ↓ thrombosis principle reflects a cytoprotective agent’s ability to normalize both extremes of bleeding and thrombosis, restoring vascular homeostasis and orderly healing [145]. Arrhythmias represent another critical facet of this systemic cytoprotection, as endothelial maintenance and vascular integrity extend pleiotropic effects from cytoprotection to organoprotection [1,2,3,4,5,6,7,8,9]. Thus, as a further conceptual point, antiarrhythmic normalization is therefore claimed as an essential component of true cytoprotection [262], and BPC 157’s anti-arrhythmic potential and its counteraction of arrhythmias has been recently reviewed [262].

Arrhythmias, particularly those impairing effective cardiac contraction, promote stasis, contributing to Virchow’s triad (stasis + endothelial dysfunction + hypercoagulability) and increasing thrombotic risk [263,264,265,266,267]. Atrial fibrillation can form left atrial appendage thrombi, while ventricular arrhythmias reduce cardiac output and favor regional or systemic hypoperfusion. Endothelial injury and arrhythmia-mediated hypercoagulability may occur even without ischemia [265,266,267].

Preclinical studies show that BPC 157 counteracts a wide array of arrhythmogenic triggers, including digitalis [268], potassium overdose [269], furosemide [270], lidocaine [271], bupivacaine [272], succinylcholine [273], neuroleptics [83,274], amphetamine [83], domperidone [83], metoclopramide [274], sotalol [84], isoprenaline [85], sodium laureate [86], alcohol [87], monocrotaline [275], hyperkalemia [269,273], hypokalemia [270], hyperlithiemia [82], and various occlusion/occlusion-like syndromes [71,72,73,74,75,76,77,78,79,80,81,82,83,84,85,86,87]. These effects also include mitigation of arrhythmias aggravated by NO/NOS modulation [85,268,269,270,271,272], supported by HEK293 studies showing direct membrane stabilization [269,270,271,272,276]. Analogous to its ability to normalize both tachycardia and bradycardia [262], BPC 157 restores hemostatic and microvascular balance without direct pro- or anticoagulant activity, integrating arrhythmia control with reduced hemorrhage and thrombosis. Notably, this could also be mobilized against a thrombus via the use of antiarrhythmics, and against hemorrhage in particular, because anticoagulants are utilized as part of an antiarrhythmic application [277,278,279,280]. Notably, as mentioned, BPC 157 is effective against sodium laurate intravenous administration [86]. This can be, as suggested, a cytoprotective therapy that preserves endothelial integrity and microvascular homeostasis [262].

Comparative pharmacology highlights BPC 157’s uniqueness. Intrinsic sympathomimetic β blockers can normalize heart rate bidirectionally [281,282,283], whereas Ca channel blockers modulate heart rate directionally—non-dihydropyridines may induce bradycardia and dihydropyridines may provoke reflex tachycardia—but lack self-normalizing capability [284,285]. ACE inhibitors and prostaglandins may modestly alter heart rate via reflex sympathetic activation [286,287,288]. NO donors or L-arginine enhance vagal tone and limit tachycardia, while NOS inhibition (L-NAME) promotes tachycardia and hypertension [289,290,291]. Statins can reduce elevated heart rates and improve autonomic balance without inducing bradycardia [292,293,294,295,296,297].

In summary, BPC 157 uniquely integrates cytoprotection across wound healing, hemostasis, thrombosis, and arrhythmias by restoring endothelial function, stabilizing cardiac electrical activity, and maintaining microvascular homeostasis. Its effects are context-dependent, bidirectional, and phase-sensitive, exemplifying the systemic principle that functional normalization—not mere inhibition or activation—underlies the resolution of hemorrhage and thrombosis.

## 5. Virchow Triad

Thrombohemorrhagic disorders, as emphasized in Section 2.7, can present with both thrombosis and hemorrhage simultaneously. Early cytoprotection studies have demonstrated that endothelial lesions, thrombus formation, and stasis precede gastric epithelial hemorrhagic lesions [4,5,6,7], implying that Virchow’s triad conditions co-occur with hemorrhage during lesion development. Consequently, cytoprotection and Virchow triad circumstances are closely linked [16,17,18,19,20,21,22,23,24,25,26], even if not explicitly framed as such initially [4,5,6,7]. As a final conceptual point, the logical extension is that cytoprotective agents, by resolving these conditions, can achieve a ↓ Virchow triad = ↓ hemorrhage + ↓ thrombosis equation, complementing the other cytoprotective principles, ↑ wound healing = ↓ hemorrhage + ↓ thrombosis and ↓ arrhythmias = ↓ hemorrhage + ↓ thrombosis [16,17,18,19,20,21,22,23,24,25,26].

BPC 157 therapy has been systematically evaluated in a broad spectrum of occlusion/occlusion-like models [71,72,73,74,75,76,77,78,79,80,81,82,83,84,85,86,87], where it consistently reversed severe vascular and multiorgan failure. Models included major vessel occlusion [74,75,76,77], bile duct occlusion with acute pancreatitis [80], intra-abdominal hypertension grade III–IV [78,79], ischemia-reperfusion injury [78,79], organ perforation [81], and exposure to cytotoxic agents such as lithium [82], intragastric alcohol [87], intravenous sodium laurate [86], isoprenaline [85], sotalol [84], neuroleptics [83], and amphetamine [83]. With BPC 157 therapy, the described ↓ Virchow triad circumstances = ↓ hemorrhage and ↓ thrombosis equation also occurs with the reversal of Pringle maneuver ischemia–reperfusion [71], Budd–Chiari syndrome models [72], and inferior caval vein ligation [73].

Restoring vascular patency, microcirculation, and endothelial integrity means that BPC 157 promptly counteracts hemorrhage and thrombosis, peripherally and centrally. This means reversing secondary lesions in the brain, heart, lung, liver, kidney, and gastrointestinal tract. Likewise, this means attenuation/elimination of various arrhythmias (bradycardias and tachycardias); intracranial, portal, and caval hypertension; and aortal hypotension. The particular reversal includes the major vessels that had failed, the inferior caval vein and superior mesenteric vein, which were congested, and the aorta and azygos vein, which were collapsed [74,75,76,77,78,79,80,81,82,83,84,85,86,87], all of which fully recovered (i.e., azygos vein activated, direct blood flow delivery). The circumstances of Virchow’s triad are regularly reversed.

A key mechanistic feature is collateral vessel recruitment, particularly the azygos vein, which rapidly redistributes venous blood when primary pathways are blocked, alleviating venous hypertension and arterial hypotension. BPC 157 also stabilizes endothelial and cardiac membranes, acts as a free radical scavenger, and maintains cellular junctions, counteracting “leaky gut” phenomena [44,145]. These vascular protective effects are closely linked to the NO system, with BPC 157 normalizing NO levels, mitigating both L-NAME-induced prothrombotic effects and L-arginine-induced hypotension/anti-coagulation [16,54,55,56,69]. Rapid vascular wall remodeling, evidenced by FTIR studies, occurs in parallel with restored perfusion, and may be responsible for the noted chain of events, commonly counteracted (i.e., resolved bradycardia or tachycardia, and reversal of peripheral and central hemorrhage and thrombosis) [298,299].

As emphasized before, these therapy effects occur even while the underlying insult persists (i.e., occlusion of major blood vessels [71,72,73,74,75,76,77], increased intra-abdominal pressure [78], grade III, grade IV) or the injury is already highly advanced and fully established [79]. This indicates a functional action at the final common pathway, whatever the molecular construct [43,44,45,46,47,48,49,50,51,52,53,54]. This explains BPC 157’s therapeutic effects, fully specified, i.e., in inferior caval vein syndrome (induced by infrarenal ligation of inferior caval vein), where there is a counteraction of the full syndrome [73]. This includes cases where there is a direct vein injury, thrombosis, thrombocytopenia, and prolonged bleeding. Rapid presentation of collaterals means instant redistribution of the trapped blood volume amid counteracted venous hypertension and arterial hypotension and tachycardia. This occurs with counteraction of oxidative stress (as a result of the lysis of endothelial cells [73]) and low NO level in the inferior caval vein. Particular gene expression (i.e., EGR, NOS, SRF, VEGR, AKT1, PLCɣ, KRAS) appears as a likely special point to explain how the dysfunction and its counteraction are causal to or a result of each other [73].

Importantly, the BPC 157 effect is conditional and context-dependent, appearing even with established and severe insults, such as complete vascular occlusion or advanced highly increased intra-abdominal pressure (grade III, grade IV), highlighting action at a final common pathway irrespective of the initial molecular insult [43,44,45,46,47,48,49,50,51,52,53,54]. Molecular studies implicate the modulation of critical signaling pathways, including VEGFR2-Akt-eNOS and Src-Caveolin-1-eNOS, supporting vasomotor tone regulation and endothelial recovery [16,45,46,47,54,55,56,67,68,69,70]. These effects occur without the need for exogenous ligands or shear stress, suggesting a direct interaction of BPC 157 with cellular targets and gasotransmitter pathways [300].

In contrast, comparative cytoprotective agents—prostaglandins, beta blockers, Ca channel blockers, ACE inhibitors, and statins—have not been tested in these occlusion/occlusion-like models. Thus, BPC 157 uniquely demonstrates a consistent ability to reverse Virchow’s triad, while simultaneously normalizing hemodynamic parameters and microvascular perfusion.

The convergence of the three cytoprotective principles—↑ wound healing, ↓ arrhythmias, and ↓ Virchow triad—suggests that BPC 157 functions as a system-level modulator, rather than a single-pathway pharmacologic agent. By integrating endothelial stabilization, collateral recruitment, NO system homeostasis, oxidative stress reduction, and vasomotor regulation, BPC 157 achieves bidirectional, phase-sensitive restoration of vascular homeostasis without pushing physiology toward either extreme [16].

This BPC 157 hemorrhage/thrombosis relation is summarized in Table 3.

**Figure 2:**
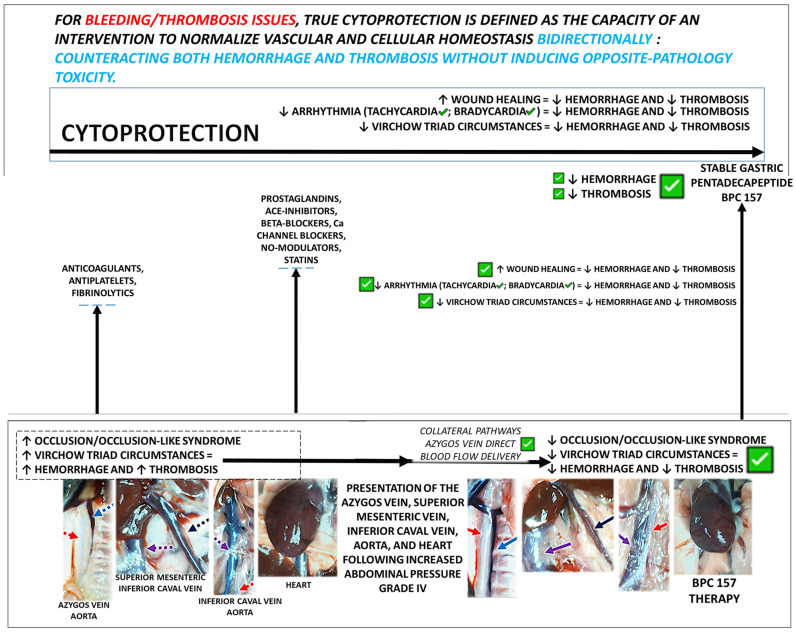
Summary of hypothesized theoretical cytoprotection framework. It is suggested that cytoprotection can be a theoretical unifying concept for the counteraction of hemorrhage and of thrombosis. Notably, as a cytoprotection mediator, BPC 157 therapy counteracts prolonged bleeding, organ hemorrhage, and thrombosis in rats, peripherally and centrally, without influencing the coagulation cascade (aggregometry and thromboelastometry studies). Counteraction of both hemorrhage and thrombosis raises a theoretical and practical query: how a useful cytoprotective therapy could support the logistical question of the “ideal agent”, also with conventional anticoagulants, antiplatelets, and fibrinolytics (≈partial cytoprotection/narrow range), and with other agents found to share some of the cytoprotective properties, prostaglandins, ACE inhibitors, beta blockers, Ca channel blockers, NO modulators, and statins (≈partial cytoprotection/more extended range). Considering hemorrhage and thrombosis, when both involved, the effects on wound healing, arrhythmias, and Virchow triad circumstances were considered as key proofs of the agent’s counteracting activity (green tick marks) on either hemorrhage or thrombosis, or the counteraction of both hemorrhage and thrombosis. Only BPC 157 demonstrates well-matched cytoprotective hemorrhage/thrombosis effects, BPC 157 ≈ full cytoprotection/wide-range homeostasis. As a concept proof ↓ hemorrhage + ↓ thrombosis, it fulfills all three converging resolving principles: **↑** Wound healing = ↓ hemorrhage + ↓ thrombosis; ↓ Arrhythmia = ↓ hemorrhage + ↓ thrombosis; ↓ Virchow triad = ↓ hemorrhage + ↓ thrombosis (see also Table 4). Nevertheless, in preclinical models, stable gastric pentadecapeptide BPC 157, in the clinic, has predominantly demonstrated a lack of adverse effects in available human trials to date. As a prominent cytoprotection mediator (LD1 not achieved in toxicology studies), it is capable of restoring vascular homeostasis, representing a novel therapeutic strategy that integrates hemostasis, endothelial protection, and functional tissue repair [16,17,18,19,20,21,22,23,24,25,26,145,262].

Likewise, being native and stable in human gastric juice (more than 24 h) while acting as a cytoprotective mediator likely allows for the translation of the maintenance of the gastrointestinal mucosa to other organ therapies, broadening the practical applicability of this peptide (i.e., pleiotropic beneficial effects, which are always applied alone without carrier), including per-oral application [16,17,18,19,20,21,22,23,24,25,26,145,262,301]. Given the consistent beneficial effects so far presented in the mentioned studies, BPC 157 therapy could specifically counteract the cytotoxic and damaging actions of NO, being organ specific. This might be seen as a network of evidence for the physiological significance of the revealed BPC 157/NO system interplay. Notably, BPC 157, native and stable in human gastric juice, was found in in situ hybridization and immunostaining studies in humans to be largely distributed in tissues [16,145,262,301] and may have additional physiological regulatory roles [16,17,18,19,20,21,22,23,24,25,26,145,301] when released from the stomach and sent to other organs. These points, and further application (i.e., efficacy and the lack of adverse effects in limited clinical trials [58,59,60,61,64,65,66]), are supported by the lack of adverse effects in toxicology studies. Notably, for BPC 157, an effective range within 10 µg–10 ng/kg means full confirmation [62,63] that a lethal dose (LD1) was not reached even at 2 g/kg i.v. or i.g. (a harmless limit test, without adverse effects in rodents) (Figure 2).

Finally, Table 4 expresses a summary of the converging resolving principles when cytoprotection was found to decrease hemorrhage and thrombosis as phase-dependent outcomes of the counteracted vascular dysregulation carried out by BPC 157 therapy.

## 6. Limitations and Future Directions

Some final notations must emphasize the complexity of the problem of the hemorrhage–thrombosis paradox as an inherent limitation for any review.

In general, there is still a dominance of one group within the presented papers [16,17,18,19,20,21,22,23,24,25,26,145,262,301]. Additionally, and in general, the cytoprotection concept [1,2,3,4,5,6,7,8,9], as a concept, is still not implemented in clinics. Taken as a hypothesis-based interpretive model (i.e., “ideal” agent capable of bidirectional regulation), it is elaborated that BPC 157 and other agents have been found to share some cytoprotective properties. Finally, decreased hemorrhage, decrease thrombosis determination goes through three converging resolving principles—wound, arrhythmias, and Virchow triad—meaning **↑** wound healing = ↓ hemorrhage + ↓ thrombosis; ↓ arrhythmia = ↓ hemorrhage + ↓ thrombosis; and ↓ Virchow triad = ↓ hemorrhage + ↓ thrombosis. All evidence combined can provide a unified systems-level model focused on BPC 157.

However, together, these could still oversimplify the multifaceted nature of the hemorrhage/thrombosis issue. Nevertheless, reliance on preclinical models necessitates further clinical validation.

Likewise, and also in general, thrombus formation and the severity, timing, and nature of blood loss—conditions that are almost impossible to standardize in clinical (human) settings—have been examined using rat models. However, it may be that a rat model simplifies these variables, which is scientifically useful but limits direct clinical predictability [302,303,304,305].

Finally, it remains to be seen whether the evidence derived from the majority of experimental work (i.e., initial discovery, mechanistic exploration, and model validation) [16,17,18,19,20,21,22,23,24,25,26,145,262,301] is found within a single research group, providing that it aligns with the confirmatory reports of other groups (i.e., [45,46,47,48,49,50,51,52,53,54,306,307,308,309,310,311,312,313]). In many studies, translational values have emphasized the similar therapeutic effect obtained by different manners of administration in the same model [16,17,18,19,20,21,22,23,24,25,26,145,262,301]. In addition to the clinical data (although still limited), studies have included favorable safety reports [58,59,60,61,62,63,64,65,66] that are comparable with previous notations [16].

There is also a need to search for a resolving analogy (preclinical models → further clinical validation). Although likely hypothetical, and not a substitute for the rigorous clinical validation that should be undertaken, such a pattern agrees with what that has been historically observed with a favorable outcome. Notably, this has already occurred, during the early preclinical phases of several now-established therapies, including the erythropoietin (EPO) [314,315] and glucagon-like peptide-1 (GLP-1) analogs before industrial expansion [316,317,318], and neuropeptides such as pituitary adenylate cyclase-activating polypeptide (PACAP) [319] and vasoactive intestinal peptide (VIP) [320,321].

## 7. Conclusions

This review addresses the hemorrhage/thrombosis ideal agent problem from a cytoprotective perspective (decreased hemorrhage and thrombosis to preserve and reestablish homeostasis). It proposes the stable gastric pentadecapeptide BPC 157, which acts as a cytoprotective mediator. In rodents, BPC 157 can simultaneously counteract hemorrhage and thrombosis without directly affecting the coagulation cascade (aggregometry, thromboelastometry). Within this framework, presenting both hemorrhage and thrombosis that should be both counteracted, the manuscript synthesizes conceptual, experimental, and clinical evidence. BPC 157’s effective wound healing, arrhythmia control, and normalization of Virchow’s triad represent converging physiological processes for the realization of the principle of decreased hemorrhage and decreased thrombosis to preserve and reestablish homeostasis.

As a comparison from a cytoprotective (partial vs. full) standpoint, conventional agents—including anticoagulants, antiplatelet drugs, fibrinolytics, beta blockers, calcium channel blockers, prostaglandins, NO modulators, ACE inhibitors, and statins—provide only partial, context-dependent protection, typically unidirectional, dose-limited, or achieved at the expense of opposing pathological risks. In contrast, preclinical evidence indicates that BPC 157 functions as a vasoprotective cytoprotective mediator capable of bidirectional regulation. Its effects likely involve the preservation of endothelial integrity, the normalization of microcirculation, the modulation of the NO system, the stabilization of hemostatic balance, and the recruitment of adaptive collateral pathways. Accordingly, with the potential limitations of conceptual points and those regarding preclinical predominance, the relationships among wound healing, arrhythmia control, and Virchow triad normalization may be viewed as interconnected cytoprotective principles contributing to the reduction of both hemorrhage and thrombosis.

Importantly, BPC 157 can counteract the bleeding induced by anticoagulants or antiplatelet agents, while simultaneously mitigating organ hemorrhage and thrombosis under conditions of vascular occlusion or arrhythmia. These properties suggest that wound healing is not merely a local reparative process, but a central organizing principle of systemic vascular homeostasis. Combined with its oral bioavailability, gastric stability, high safety profile, and efficacy in both prophylactic and therapeutic settings, BPC 157 holds substantial translational potential as a novel cytoprotective strategy for complex vascular and hemostatic disorders.

## Figures and Tables

**Table 1:** Summary including anticoagulants, antiplatelets, fibrinolytics, and BPC 157, highlighting their effects on thrombosis and bleeding and how they “tip the hemostatic balance”.

Drug/Class	Primary Effect on Thrombosis	Primary Effect on Bleeding	Mechanistic Comment	Cytoprotective Aspect/Notes
**Anticoagulants** (heparin, LMWH, DOACs, warfarin)	↓ Thrombosis (prevents clot formation)	↑ Bleeding risk	Strong inhibition of the coagulation cascade; warfarin may cause vascular calcification (loss of vitamin K-dependent protective proteins)	Some (DOACs, heparin) show endothelial protection, anti-inflammatory and antioxidant effects; warfarin lacks cytoprotection
**Antiplatelets** (aspirin, P2Y12 inhibitors)	↓ Arterial thrombosis	↑ Bleeding tendency	Inhibit platelet aggregation; risk of rebound platelet hyperreactivity after withdrawal	Aspirin shows endothelial-protective and antioxidant effects; P2Y12 inhibitors reduce ischemia-reperfusion injury and neutrophil-mediated endothelial damage
**Fibrinolytics** (tPA, streptokinase, urokinase)	↓ Existing thrombi	↑ Bleeding risk	Lyse clots but may expose procoagulant surfaces; risk of acute rethrombosis (“thrombolytic paradox”)	Indirect cytoprotection via restored perfusion; limits apoptosis, oxidative stress, and secondary inflammation
**BPC 157**	↓ Thrombosis (counteracts occlusion/ occlusion-like syndromes)	↓ Hemorrhage (counteracts prolonged bleeding)	Multi-pathway vascular and tissue protection; stabilizes endothelial function and platelet–vessel interactions	Truly cytoprotective: normalizes hemostasis, vascular integrity, and tissue repair without tipping the balance

**Table 2:** Comparative effects of other cytoprotective/pharmacologic agents.

Agent	Effect on Wound Healing	Effect on Hemorrhage	Effect on Thrombosis	Notes
**Prostaglandins (PGE1, PGE2,** **iloprost)**	↑ microcirculation, ↑ healing in burns/ulcers	May increase bleeding	Prostacyclin analogs prevent thrombosis in PAD/dialysis grafts	Excessive PGE2 may promote inflammation/scarring [195]
**Statins**	Low/moderate doses: pro-healing; high doses: delayed repair	↓ bleeding risk in anticoagulated patients [235]	↓ venous thromboembolism risk [236]	Dose-dependent effects; pro-healing via angiogenesis modulation [196]
**ACE inhibitors**	↑ wound healing [197,198]	↑ bleeding risk in some contexts [223,224]	Neutral or anti-thrombotic in CV trials [225,226]	Rare thrombosis: context dependent [228,229]
**Beta blockers**	↑ or ↓ wound healing depending on study and dose [200,201]	↓ bleeding in cirrhosis/varices [230,231]	Reduced coronary flow may increase thrombosis [232]	Biphasic and context-dependent effects
**Ca-channel blockers**	↑ wound healing (topical nifedipine, amlodipine, verapamil) [205]	↑ bleeding mainly with anticoagulants [233]	Dihydropyridine CCBs associated with ischemic stroke in AF patients [234]	Clinical context critical
**NO donors**	↑ wound healing, angiogenesis, fibroblast function [212,213]	↑ bleeding if systemic/high dose	Endogenous NO: anti-platelet, anti-thrombotic; NOS inhibition ↑ thrombosis [238]	Phase-dependent therapy crucial

**Table 3:** Summary of counteraction of hemorrhage and thrombosis by stable gastric pentadecapeptide BPC 157 in rats. Notably, as a common point, in occlusion/occlusion-like syndromes [74,75,76,77,78,79,80,81,82,83,84,85,86,87] counteraction includes the severe lesions in the brain, heart, lung, liver, kidney, and gastrointestinal tract; attenuation/elimination of various arrhythmias; intracranial, portal, and caval hypertension; and aortal hypotension. Attenuation/elimination occurs with prompt attenuation/elimination of hemorrhage and thrombosis occurring peripherally and centrally. The particular reversal includes the major vessels that had failed—the inferior caval vein and the superior mesenteric vein, which were congested, and the aorta and azygos vein, which were collapsed [74,75,76,77,78,79,80,81,82,83,84,85,86,87]—and which all fully recovered (i.e., azygos vein activated, direct blood flow delivery).

Experimental Model	Hemorrhagic/Thrombotic Challenge	Key Vascular/Hemostatic Outcomes	Main Conclusion
Abdominal aorta anastomosis (rat)	Surgical vascular injury, thrombosis risk	Improved anastomosis patency, reduced thrombosis, preserved endothelium	BPC 157 counteracts thrombus formation, and any established thrombus is annihilated, recovering vascular protection [67]
Limb amputation + anticoagulants	Heparin-, warfarin-, aspirin-induced bleeding	Shortened bleeding time, reduced thrombocytopenia	BPC 157 counteracts anticoagulant- and antiplatelet-induced hemorrhage [68]
Amputation + NO system manipulation	Heparin, warfarin, L-NAME, L-arginine	Reduced bleeding, restored platelet count, NO system balance	Hemostasis restored despite anticoagulation and NO dysregulation [69]
Antiplatelet drugs (intragastric)	Aspirin, clopidogrel, cilostazol	Normalized platelet aggregation and clot formation	BPC 157 stabilizes platelet–clot function [70]
Pringle maneuver	Hepatic ischemia–reperfusion, venous congestion	Reduced hemorrhage, and thrombosis, restored hepatic circulation	Rapid collateral recruitment counteracts vascular failure [71]
Budd–Chiari syndrome	Suprahepatic IVC occlusion, thrombosis	Resolution of portal/caval hypertension, reduced bleeding, and reduced thrombosis	Recovered Budd–Chiari syndrome Thrombotic venous occlusion reversed [72]
Inferior caval vein ligation	Venous thrombosis, congestion	Activation of collateral pathways counteracted stasis	BPC 157 resolves venous thrombosis-like states [73]
Superior mesenteric vein occlusion	Mesenteric thrombosis, ischemia	Reduced hemorrhage, intestinal protection, reduced bleeding, and reduced thrombosis	Venous occlusion counteracted systemically [74]
Superior mesenteric vein occlusion + superior mesenteric artery occlusion	Combined arterial–venous thrombosis	Restored perfusion, reduced organ bleeding, and reduced thrombosis	Counteracted the consequences of the simultaneous occlusion of a major artery and vein [75]
Superior mesenteric artery occlusion	Arterial, venous thrombosis, hypotension	Restored perfusion, reduced organ bleeding, and reduced thrombosis	Arterial occlusion reversed without bleeding [76]
Superior sagittal sinus occlusion	Cerebral venous thrombosis	Collateral sinus recruitment, reduced brain edema Restored perfusion, reduced organ bleeding, and reduced thrombosis	Counteracted the consequences of central occlusion, both centrally and peripherally [77]
Abdominal compartment syndrome, grade III, IV	Compression-induced vascular failure	Normalized venous return Restored perfusion, reduced organ bleeding, and reduced thrombosis	Mechanical vascular collapse reversed [78]
Intra-abdominal hypertension grade III, IV + reperfusion	Ischemia/reperfusion bleeding risk	Improved reperfusion, reduced hemorrhagic injury, and reduced thrombosis	Endothelial stabilization during reperfusion [79]
Acute pancreatitis	Microvascular thrombosis, bleeding	Reduced pancreatic hemorrhage, restored flow Reduced other organ bleeding, reduced thrombosis	Vascular failure as primary target [80]
Stomach perforation	General occlusion/occlusion-like syndrome	Hemodynamic stabilization, reduced bleeding	Systemic vascular rescue [81]
Lithium overdose	Occlusion-like vascular syndrome	Reduced hemorrhage, restored circulation, reduced thrombosis	Toxicity-associated hemorrhage and thrombosis counteracted [82]
Neuroleptics/ amphetamine/ domperidone	Drug-induced vascular collapse	Reduced hemorrhage, restored circulation, reduced thrombosis	Innate vascular failure normalized [83]
Sotalol-induced arrhythmia	Occlusion-like syndrome, hypotension	Antiarrhythmic + vascular stabilization Reduced hemorrhage, restored circulation, reduced thrombosis	Links arrhythmia, thrombosis, and cytoprotection [84]
Isoprenaline-induced myocardial infarction	Coronary thrombosis, hemorrhagic necrosis	Reduced infarct size, preserved vessels Reduced hemorrhage, restored circulation, reduced thrombosis	Cardiovascular thrombohemorrhagic balance [85]
Inferior caval vein embolization	Embolism, post-embolization syndrome	Rapid resolution of embolic consequences Reduced hemorrhage, restored circulation, reduced thrombosis	Safe embolus counteraction [86]
Alcohol-induced gastric lesions	Hemorrhagic mucosal injury	Reduced hemorrhage, restored circulation, reduced thrombosis	Hemorrhage/thrombosis counteracted via cytoprotection, given alcohol as a key cytoprotection model [87]

**Table 4:** Converging resolving principles of BPC 157 therapy.

Resolving Principle	Mechanistic Basis	Key Effects/Observations	Outcome
**↑ Wound Healing**	Tissue repair, endothelial maintenance, angiogenesis, collagen deposition, microvascular restoration	Promotes rapid hemostasis, clot resolution, anastomosis healing, tendon/muscle repair, corneal ulcer recovery	↓ Hemorrhage, ↓ Thrombosis
**↓ Arrhythmias**	Membrane stabilization, autonomic balance, NO system modulation	Counteracts tachycardia and bradycardia, protects cardiac conduction, preserves cardiac output	↓ Hemorrhage, ↓ Thrombosis
**↓ Virchow Triad**	Restoration of endothelial integrity, collateral vessel recruitment (e.g., azygos vein), reduction of stasis and hypercoagulability	Resolves arterial/venous occlusion, ischemia–reperfusion injury, drug-induced occlusion/occlusion-like syndromes	↓ Hemorrhage, ↓ Thrombosis
**System-Level** **Integration**	Combined action on wound healing, arrhythmias, and Virchow triad	Maintains NO system homeostasis, stabilizes microcirculation, prevents organ failure	Restored vascular homeostasis; bidirectional hemostatic balance

## Data Availability

No new data were created or analyzed in this study. Data sharing is not applicable to this article.

## References

[1] Robert A. (1979). Cytoprotection by prostaglandins. Gastroenterology.

[2] Robert A. (1981). Current history of cytoprotection. Prostaglandins.

[3] Robert A., Nezamis J.E., Lancaster C., Davis J.P., Field S.O., Hanchar A.J. (1983). Mild irritants prevent gastric necrosis through “adaptive cytoprotection” mediated by prostaglandins. Am. J. Physiol..

[4] Szabo S. (1984). Role of sulfhydryls and early vascular lesions in gastric mucosal injury. Acta Physiol. Hung..

[5] Trier J.S., Szabo S., Allan C.H. (1987). Ethanol-induced damage to mucosal capillaries of rat stomach. Ultrastructural features and effects of prostaglandin F2β and cysteamine. Gastroenterology.

[6] Pihan G., Majzoubi D., Haudenschild C., Trier J.S., Szabo S. (1986). Early microcirculatory stasis in acute gastric mucosal injury in the rat and prevention by 16,16-dimethyl prostaglandin E2 or sodium thiosulfate. Gastroenterology.

[7] Szabo S., Trier J.S., Brown A., Schnoor J. (1985). Early vascular injury and increased vascular permeability in gastric mucosal injury caused by ethanol in the rat. Gastroenterology.

[8] Szabo S. (1986). Experimental basis for a role for sulfhydryls and dopamine in ulcerogenesis: A primer for cytoprotection-organoprotection. Klin. Wochenschr..

[9] Szabo S., Usadel K.H. (1982). Cytoprotection-organoprotection by somatostatin: Gastric and hepatic lesions. Experientia.

[10] Gale A.J. (2011). Continuing education course #2: Current understanding of hemostasis. Toxicol. Pathol..

[11] Furie B., Furie B.C. (2008). Mechanisms of thrombus formation. N. Engl. J. Med..

[12] Linkins L.-A., Choi P.T., Douketis J.D. (2003). Clinical impact of bleeding in patients taking oral anticoagulant therapy for venous thromboembolism. Ann. Intern. Med..

[13] Levi M., Levy J.H., Andersen H.F., Truloff D. (2010). Safety of recombinant activated factor VII in randomized clinical trials. N. Engl. J. Med..

[14] Hoffman M., Monroe D.M. (2001). A cell-based model of hemostasis. Thromb. Haemost..

[15] Mackman N. (2008). Triggers, targets and treatments for thrombosis. Nature.

[16] Sikiric P., Seiwerth S., Skrtic A., Staresinic M., Strbe S., Vuksic A., Sikiric S., Bekic D., Soldo D., Grizelj B. (2025). Stable gastric pentadecapeptide BPC 157 as a therapy and safety key: A special beneficial pleiotropic effect controlling and modulating angiogenesis and the NO-system. Pharmaceuticals.

[17] Sikiric P., Seiwerth S., Skrtic A., Staresinic M., Strbe S., Vuksic A., Sikiric S., Bekic D., Penovic T., Drazenovic D. (2025). Acute compartment syndrome and intra-abdominal hypertension, decompression, current pharmacotherapy, and stable gastric pentadecapeptide BPC 157 solution. Pharmaceuticals.

[18] Sikiric P., Sever M., Krezic I., Vranes H., Kalogjera L., Smoday I.M., Vukovic V., Oroz K., Coric L., Skoro M. (2024). New studies with stable gastric pentadecapeptide protecting gastrointestinal tract. Significance of counteraction of vascular and multiorgan failure of occlusion/occlusion-like syndrome in cytoprotection/organoprotection. Inflammopharmacology.

[19] Sikiric P., Boban Blagaic A., Strbe S., Beketic Oreskovic L., Oreskovic I., Sikiric S., Staresinic M., Sever M., Kokot A., Jurjevic I. (2024). The stable gastric pentadecapeptide BPC 157 pleiotropic beneficial activity and its possible relations with neurotransmitter activity. Pharmaceuticals.

[20] Sikiric P., Gojkovic S., Knezevic M., Tepes M., Strbe S., Vukojevic J., Duzel A., Kralj T., Krezic I., Zizek H. (2023). Stable gastric pentadecapeptide BPC 157: Prompt particular activation of collateral pathways. Curr. Med. Chem..

[21] Sikiric P., Kokot A., Kralj T., Zlatar M., Masnec S., Lazic R., Loncaric K., Oroz K., Sablic M., Boljesic M. (2023). Stable gastric pentadecapeptide BPC 157—Possible novel therapy of glaucoma and other ocular conditions. Pharmaceuticals.

[22] Staresinic M., Japjec M., Vranes H., Prtoric A., Zizek H., Krezic I., Gojkovic S., Smoday I.M., Oroz K., Staresinic E. (2022). Stable gastric pentadecapeptide BPC 157 and striated, smooth, and heart muscle. Biomedicines.

[23] Sikiric P., Udovicic M., Barisic I., Balenovic D., Zivanovic Posilovic G., Strinic D., Uzun S., Sikiric S., Krezic I., Zizek H. (2022). Stable gastric pentadecapeptide BPC 157 as useful cytoprotective peptide therapy in the heart disturbances, myocardial infarction, heart failure, pulmonary hypertension, arrhythmias, and thrombosis presentation. Biomedicines.

[24] Sikiric P., Skrtic A., Gojkovic S., Krezic I., Zizek H., Lovric E., Sikiric S., Knezevic M., Strbe S., Milavic M. (2022). Cytoprotective gastric pentadecapeptide BPC 157 resolves major vessel occlusion disturbances, ischemia-reperfusion injury following Pringle maneuver, and Budd-Chiari syndrome. World J. Gastroenterol..

[25] Sikiric P., Seiwerth S., Skrtic A., Staresinic M., Strbe S., Vuksic A., Sikiric S., Bekic D., Soldo D., Grizelj B. (2025). BPC 157 Therapy: Targeting angiogenesis and nitric oxide’s cytotoxic and damaging actions, but maintaining, promoting, or recovering their essential protective functions. Comment on Józwiak et al. Multifunctionality and Possible Medical Application of the BPC 157 Peptide—Literature and Patent Review. Pharmaceuticals.

[26] Masnec S., Kokot A., Kralj T., Zlatar M., Loncaric K., Sablic M., Kalauz M., Beslic I., Oroz K., Mrvelj B. (2025). Challenge of corneal ulcer healing: A novel conceptual framework, the “triad” of corneal ulcer healing/corneal neovascularization/intraocular pressure, and avascular tendon healing, for evaluation of corneal ulcer therapy, therapy of neovascularization, glaucoma therapy, and pentadecapeptide BPC 157 efficacy. Pharmaceuticals.

[27] Niki E., Noguchi N., Tsuchihashi H., Gotoh N. (1995). Interaction among vitamin C, vitamin E, and β-carotene. Am. J. Clin. Nutr..

[28] Liguori I., Russo G., Curcio F., Bulli G., Aran L., Della-Morte D., Gargiulo G., Testa G., Cacciatore F., Bonaduce D. (2018). Oxidative stress, aging, and diseases. Clin. Interv. Aging.

[29] Belenichev I., Popazova O., Bukhtiyarova N., Savchenko D., Oksenych V., Kamyshnyi O. (2024). Modulating nitric oxide: Implications for cytotoxicity and cytoprotection. Antioxidants.

[30] Shahin Y., Khan J.A., Samuel N., Chetter I. (2011). Angiotensin converting enzyme inhibitors effect on endothelial dysfunction: A meta-analysis of randomised controlled trials. Atherosclerosis.

[31] Liao J.K., Laufs U. (2005). Pleiotropic effects of statins. Annu. Rev. Pharmacol. Toxicol..

[32] Davignon J. (2004). Beneficial cardiovascular pleiotropic effects of statins. Circulation.

[33] Xu C., Hu Y., Hou L., Ju J., Li X., Du N., Guan X., Liu Z., Zhang T., Qin W. (2014). β-Blocker carvedilol protects cardiomyocytes against oxidative stress-induced apoptosis by up-regulating miR-133 expression. J. Mol. Cell. Cardiol..

[34] Nakamura K., Murakami M., Miura D., Yunoki K., Enko K., Tanaka M., Saito Y., Nishii N., Miyoshi T., Yoshida M. (2011). Beta-blockers and oxidative stress in patients with heart failure. Pharmaceuticals.

[35] Mason R.P., Mak I.T., Walter M.F., Tulenko T.N., Mason P.E. (1998). Antioxidant and cytoprotective activities of the calcium channel blocker mibefradil. Biochem. Pharmacol..

[36] Deakin C.D., Fagan E.A., Williams R. (1991). Cytoprotective effects of calcium channel blockers: Mechanisms and potential applications in hepatocellular injury. J. Hepatol..

[37] Evrova O., Kellenberger D., Calcagni M., Vogel V., Buschmann J. (2020). Supporting cell-based tendon therapy: Effect of PDGF-BB and ascorbic acid on rabbit Achilles tenocytes in vitro. Int. J. Mol. Sci..

[38] Kaji D.A., Howell K.L., Balic Z., Hubmacher D., Huang A.H. (2020). TGFβ signaling is required for tenocyte recruitment and functional neonatal tendon regeneration. eLife.

[39] Lu J., Jiang L., Chen Y., Lyu K., Zhu B., Li Y., Liu X., Liu X., Long L., Wang X. (2022). The functions and mechanisms of basic fibroblast growth factor in tendon repair. Front. Physiol..

[40] Liang M., Cornell H.R., Zargar Baboldashti N., Thompson M.S., Carr A.J., Hulley P.A. (2012). Regulation of hypoxia-induced cell death in human tenocytes. Adv. Orthop..

[41] Scott A., Khan K.M., Duronio V. (2005). IGF-I activates PKB and prevents anoxic apoptosis in Achilles tendon cells. J. Orthop. Res..

[42] Li Z., Shen X., Cao L., Yuan Z., Chen S., Zheng X., Tang M., Lee K.K., Cai D. (2011). Bone morphogenetic protein 2 improves patellar tendon healing by promoting migration and proliferation of tenocytes. Chin. Sci. Bull..

[43] Kang E.A., Han Y.M., An J.M., Park Y.J., Sikiric P., Kim D.H., Kwon K.A., Kim Y.J., Yang D., Tchah H. (2018). BPC157 as potential agent rescuing from cancer cachexia. Curr. Pharm. Des..

[44] Park J.M., Lee H.J., Sikiric P., Hahm K.B. (2020). BPC157 rescued NSAID-cytotoxicity via stabilizing intestinal permeability and enhancing cytoprotection. Curr. Pharm. Des..

[45] Wu H., Wei M., Li N., Lu Q., Shrestha S.M., Tan J., Zhang Z., Wu G., Shi R. (2020). Clopidogrel-induced gastric injury in rats is attenuated by stable gastric pentadecapeptide BPC 157. Drug Des. Dev. Ther..

[46] Hsieh M.J., Lee C.H., Chueh H.Y., Chang G.J., Huang H.Y., Lin Y., Pang J.H.S. (2020). Modulatory effects of BPC 157 on vasomotor tone and the activation of Src–caveolin-1–endothelial nitric oxide synthase pathway. Sci. Rep..

[47] Hsieh M.J., Liu H.T., Wang C.N., Huang H.Y., Lin Y., Ko Y.S., Wang J.S., Chang V.H.S., Pang J.H.S. (2017). Therapeutic potential of pro-angiogenic BPC157 is associated with VEGFR2 activation and up-regulation. J. Mol. Med..

[48] Huang B.S., Huang S.C., Chen F.H., Chang Y., Mei H.F., Huang H.Y., Chen W.Y., Pang J.S. (2022). Pentadecapeptide BPC 157 efficiently reduces radiation-induced liver injury and lipid accumulation through Kruppel-like factor 4 upregulation both in vivo and in vitro. Life Sci..

[49] Wang X.Y., Qu M., Duan R., Shi D., Jin L., Gao J., Wood J.D., Li J., Wang G.D. (2019). Cytoprotective mechanism of the novel gastric peptide BPC157 in gastrointestinal tract and cultured enteric neurons and glial cells. Neurosci. Bull..

[50] Chang C.H., Tsai W.C., Hsu Y.H., Pang J.H.S. (2014). Pentadecapeptide BPC 157 enhances the growth hormone receptor expression in tendon fibroblasts. Molecules.

[51] Chang C.H., Tsai W.C., Lin M.S., Hsu Y.H., Pang J.H.S. (2011). The promoting effect of pentadecapeptide BPC 157 on tendon healing involves tendon outgrowth, cell survival, and cell migration. J. Appl. Physiol..

[52] Radeljak S., Seiwerth S., Sikiric P. (2004). BPC 157 inhibits cell growth and VEGF signalling via the MAPK kinase pathway in the human melanoma cell line. Melanoma Res..

[53] Ivetić Tkalcević V., Cuzić S., Brajsa K., Mildner B., Bokulić A., Situm K., Perović D., Glojnarić I., Parnham M.J. (2007). Enhancement by PL 14736 of granulation and collagen organization in healing wounds and the potential role of egr-1 expression. Eur. J. Pharmacol..

[54] Huang T., Zhang K., Sun L., Xue X., Zhang C., Shu Z., Mu N., Gu J., Zhang W., Wang Y. (2015). Body protective compound-157 enhances alkali-burn wound healing in vivo and promotes proliferation, migration, and angiogenesis in vitro. Drug Des. Devel. Ther..

[55] Sikirić P., Seiwerth S., Grabarević Z., Rucman R., Petek M., Jagić V., Turković B., Rotkvić I., Miše S., Zoričić I. (1997). The influence of a novel pentadecapeptide, BPC 157, on N(G)-nitro-L-arginine methyl ester- and L-arginine-induced effects on stomach mucosa integrity and blood pressure. Eur. J. Pharmacol..

[56] Turković B., Sikirić P., Seiwerth S., Miše S., Aničić T., Petek M. (2004). Stable gastric pentadecapeptide BPC 157 studied for inflammatory bowel disease (PLD-116, PL-14736, Pliva) induces nitric oxide synthesis. Gastroenterology.

[57] Sikiric P., Drmic D., Boban Blagaic A., Tvrdeic A., Krezic I., Gojkovic S., Zizek H., Sikiric S., Strbe S., Smoday I.M., Ray A., Gulati K. (2023). Stable gastric pentadecapeptide BPC 157 and the nitric oxide system. Nitric Oxide: From Research to Therapeutics; Advances in Biochemistry in Health and Disease.

[58] Ruenzi M., Stolte M., Veljaca M., Oreskovic K., Peterson J. (2005). Ulcerative Colitis Study Group. A multicenter, randomized, double blind, placebo-controlled phase II study of PL 14736 enema in the treatment of mild-to-moderate ulcerative colitis. Gastroenterology.

[59] Veljaca M., Pavic-Sladoljev D., Mildner B., Brajsa K., Bubenik M., Stipanicic S., Parnham M. (2003). Safety, tolerability and pharmacokinetics of PL14736, a novel agent for treatment of ulcerative colitis, in healthy male volunteers. Gut.

[60] Lee E., Padgett B. (2021). Intra-articular injection of BPC 157 for multiple types of knee pain. Altern. Ther. Health Med..

[61] Lee E., Walker C., Ayadi B. (2024). Effect of BPC-157 on symptoms in patients with interstitial cystitis: A pilot study. Altern. Ther. Health Med..

[62] He L., Feng D., Guo H., Zhou Y., Li Z., Zhang K., Zhang W., Wang S., Wang Z., Hao Q. (2022). Pharmacokinetics, distribution, metabolism, and excretion of body-protective compound 157, a potential drug for treating various wounds, in rats and dogs. Front. Pharmacol..

[63] Xu C., Sun L., Ren F., Huang P., Tian Z., Cui J., Zhang W., Wang S., Zhang K., He L. (2020). Preclinical safety evaluation of body protective compound-157, a potential drug for treating various wounds. Regul. Toxicol. Pharmacol..

[64] Lee E., Burgess K. (2025). Safety of intravenous infusion of BPC157 in humans: A pilot study. Altern. Ther. Health Med..

[65] (2024). October 29, 2024: Meeting of the Pharmacy Compounding Advisory Committee. https://www.fda.gov/advisory-committees/advisory-committee-calendar/october-29-2024-meeting-pharmacy-compounding-advisory-committee-10292024.

[66] (2024). Updated Meeting Time and Public Participation Information. December 4, 2024: Meeting of the Pharmacy Compounding Advisory Committee. https://www.fda.gov/advisory-committees/advisory-committee-calendar/updated-meeting-time-and-public-participation-information-december-4-2024-meeting-pharmacy.

[67] Hrelec M., Klicek R., Brcic L., Brcic I., Cvjetko I., Seiwerth S., Sikiric P. (2009). Abdominal aorta anastomosis in rats and stable gastric pentadecapeptide BPC 157, prophylaxis and therapy. J. Physiol. Pharmacol..

[68] Stupnisek M., Franjic S., Drmic D., Hrelec M., Kolenc D., Radic B., Bojic D., Vcev A., Seiwerth S., Sikiric P. (2012). Pentadecapeptide BPC 157 reduces bleeding time and thrombocytopenia after amputation in rats treated with heparin, warfarin or aspirin. Thromb. Res..

[69] Stupnisek M., Kokot A., Drmic D., Hrelec Patrlj M., Zenko Sever A., Kolenc D., Radic B., Suran J., Bojic D., Vcev A. (2015). Pentadecapeptide BPC 157 reduces bleeding and thrombocytopenia after amputation in rats treated with heparin, warfarin, L-NAME and L-arginine. PLoS ONE.

[70] Konosic S., Petricevic M., Ivancan V., Konosic L., Goluza E., Krtalic B., Drmic D., Stupnisek M., Seiwerth S., Sikiric P. (2019). Intragastric application of aspirin, clopidogrel, cilostazol, and BPC 157 in rats: Platelet aggregation and blood clot. Oxid. Med. Cell. Longev..

[71] Kolovrat M., Gojković S., Krezić I., Malekinušić D., Vrdoljak B., Kašnik Kovač K., Kralj T., Drmić D., Barišić I., Horvat Pavlov K. (2020). Pentadecapeptide BPC 157 resolves Pringle maneuver in rats, both ischemia and reperfusion. World J. Hepatol..

[72] Gojković S., Krezić I., Vrdoljak B., Malekinušić D., Barišić I., Petrović A., Horvat Pavlov K., Kolovrat M., Duzel A., Knežević M. (2020). Pentadecapeptide BPC 157 resolves suprahepatic occlusion of the inferior caval vein, Budd–Chiari syndrome model in rats. World J. Gastrointest. Pathophysiol..

[73] Vukojevic J., Siroglavic M., Kasnik K., Kralj T., Stancic D., Kokot A., Kolaric D., Drmic D., Zenko Sever A., Barisic I. (2018). Rat inferior caval vein (ICV) ligature and particular new insights with the stable gastric pentadecapeptide BPC 157. Vascul. Pharmacol..

[74] Knezevic M., Gojkovic S., Krezic I., Zizek H., Vranes H., Malekinusic D., Vrdoljak B., Knezevic T., Horvat Pavlov K., Drmic D. (2021). Complex syndrome of the complete occlusion of the end of the superior mesenteric vein, opposed with the stable gastric pentadecapeptide BPC 157 in rats. Biomedicines.

[75] Knezevic M., Gojkovic S., Krezic I., Zizek H., Malekinusic D., Vrdoljak B., Knezevic T., Vranes H., Drmic D., Staroveski M. (2021). Occluded superior mesenteric artery and vein. Therapy with the stable gastric pentadecapeptide BPC 157. Biomedicines.

[76] Knezevic M., Gojkovic S., Krezic I., Zizek H., Malekinusic D., Vrdoljak B., Vranes H., Knezevic T., Barisic I., Horvat Pavlov K. (2021). Occlusion of the superior mesenteric artery in rats reversed by collateral pathways activation: Gastric pentadecapeptide BPC 157 therapy counteracts multiple organ dysfunction syndrome; intracranial, portal, and caval hypertension; and aortal hypotension. Biomedicines.

[77] Gojkovic S., Krezic I., Vranes H., Zizek H., Drmic D., Horvat Pavlov K., Petrovic A., Batelja Vuletic L., Milavic M., Sikiric S. (2021). BPC 157 therapy and the permanent occlusion of the superior sagittal sinus in rat: Vascular recruitment. Biomedicines.

[78] Tepes M., Gojkovic S., Krezic I., Zizek H., Vranes H., Madzar Z., Santak G., Batelja L., Milavic M., Sikiric S. (2021). Stable gastric pentadecapeptide BPC 157 therapy for primary abdominal compartment syndrome in rats. Front. Pharmacol..

[79] Tepes M., Krezic I., Vranes H., Smoday I.M., Kalogjera L., Zizek H., Vukovic V., Oroz K., Kasnik Kovac K., Madzar Z. (2023). Stable gastric pentadecapeptide BPC 157 therapy: Effect on reperfusion following maintained intra-abdominal hypertension (grade III and IV) in rats. Pharmaceuticals.

[80] Smoday I.M., Petrovic I., Kalogjera L., Vranes H., Zizek H., Krezic I., Gojkovic S., Skorak I., Hriberski K., Brizic I. (2022). Therapy effect of the stable gastric pentadecapeptide BPC 157 on acute pancreatitis as vascular failure-induced severe peripheral and central syndrome in rats. Biomedicines.

[81] Kalogjera L., Krezic I., Smoday I.M., Vranes H., Zizek H., Yago H., Oroz K., Vukovic V., Kavelj I., Novosel L. (2023). Stomach perforation-induced general occlusion/occlusion-like syndrome and stable gastric pentadecapeptide BPC 157 therapy effect. World J. Gastroenterol..

[82] Strbe S., Gojkovic S., Krezic I., Zizek H., Vranes H., Barisic I., Strinic D., Orct T., Vukojevic J., Ilic S. (2021). Over-dose lithium toxicity as an occlusive-like syndrome in rats and gastric pentadecapeptide BPC 157. Biomedicines.

[83] Strbe S., Smoday I.M., Krezic I., Kalogjera L., Vukovic V., Zizek H., Gojkovic S., Vranes H., Barisic I., Sikiric S. (2023). Innate vascular failure by application of neuroleptics, amphetamine, and domperidone rapidly induced severe occlusion/occlusion-like syndromes in rats and stable gastric pentadecapeptide BPC 157 as therapy. Pharmaceuticals.

[84] Premuzic Mestrovic I., Smoday I.M., Kalogjera L., Krezic I., Zizek H., Vranes H., Vukovic V., Oroz K., Skorak I., Brizic I. (2023). Antiarrhythmic sotalol, occlusion/occlusion-like syndrome in rats, and stable gastric pentadecapeptide BPC 157 therapy. Pharmaceuticals.

[85] Barisic I., Balenovic D., Udovicic M., Bardak D., Strinic D., Vlainić J., Vranes H., Smoday I.M., Krezic I., Milavic M. (2022). Stable gastric pentadecapeptide BPC 157 may counteract myocardial infarction induced by isoprenaline in rats. Biomedicines.

[86] Smoday I.M., Krezic I., Kalogjera L., Vukovic V., Zizek H., Skoro M., Kasnik Kovac K., Vranes H., Barisic I., Sikiric S. (2023). Pentadecapeptide BPC 157 as therapy for inferior caval vein embolization: Recovery of sodium laurate-post-embolization syndrome in rats. Pharmaceuticals.

[87] Gojkovic S., Krezic I., Vranes H., Zizek H., Drmic D., Batelja Vuletic L., Milavic M., Sikiric S., Stilinovic I., Simeon P. (2021). Robert’s intragastric alcohol-induced gastric lesion model as an escalated general peripheral and central syndrome, counteracted by the stable gastric pentadecapeptide BPC 157. Biomedicines.

[88] Sikiric P., Seiwerth S., Rucman R., Turkovic B., Rokotov D.S., Brcic L., Sever M., Klicek R., Radic B., Drmic D. (2013). Toxicity by NSAIDs. Counteraction by stable gastric pentadecapeptide BPC 157. Curr. Pharm. Des..

[89] Atzemian N., Kareli D., Ragia G., Manolopoulos V.G. (2023). Distinct pleiotropic effects of direct oral anticoagulants on cultured endothelial cells: A comprehensive review. Front. Pharmacol..

[90] Alfano F., Gori A.M., Berteotti M., Rogolino A., Cesari F., Salvadori E., Formelli B., Pescini F., Barbato C., Giusti B. (2025). Pleiotropic effects of oral anticoagulant therapy: Is there a difference between VKAs and DOACs?. Biomedicines.

[91] Puech C., Delavenne X., He Z., Forest V., Mismetti P., Perek N. (2019). Direct oral anticoagulants are associated with limited damage of endothelial cells of the blood-brain barrier mediated by the thrombin/PAR-1 pathway. Brain Res..

[92] Gikakis N., Khan M.M., Hiramatsu Y., Gorman J.H., Hack C.E., Sun L., Rao A.K., Niewiarowski S., Colman R.W., Edmunds L.H. (1996). Effect of factor Xa inhibitors on thrombin formation and complement and neutrophil activation during in vitro extracorporeal circulation. Circulation.

[93] Ten Cate H., Guzik T.J., Eikelboom J., Spronk H.M.H. (2021). Pleiotropic actions of factor Xa inhibition in cardiovascular prevention: Mechanistic insights and implications for anti-thrombotic treatment. Cardiovasc. Res..

[94] Bal Dit Sollier C., Dillinger J.-G., Drouet L. (2020). Anticoagulant activity and pleiotropic effects of heparin. J. Med. Vasc..

[95] Price P.A., Faus S.A., Williamson M.K. (1998). Warfarin causes rapid calcification of the elastic lamellae in rat arteries and heart valves. Arterioscler. Thromb. Vasc. Biol..

[96] Schurgers L.J., Spronk H.M.H., Skepper J.N., Hackeng T.M., Shanahan C.M., Vermeer C., Weissberg P.L., Proudfoot D. (2007). Post-translational modifications regulate matrix Gla protein function: Importance for inhibition of vascular smooth muscle cell calcification. J. Thromb. Haemost..

[97] Zhai X., Pu D., Wang R., Zhang J., Lin Y., Wang Y., Zhai N., Peng X., Zhou Q., Li L. (2023). Gas6/AXL pathway: Immunological landscape and therapeutic potential. Front. Oncol..

[98] Fraineau S., Monvoisin A., Clarhaut J., Talbot J., Simonneau C., Kanthou C., Kanse S.M., Philippe M., Benzakour O. (2012). The vitamin K–dependent anticoagulant factor, protein S, inhibits multiple VEGF-A–induced angiogenesis events in a Mer- and SHP2-dependent manner. Blood.

[99] Barrett H., O’Keeffe M., Kavanagh E., Walsh M., O’Connor E.M. (2018). Is matrix Gla protein associated with vascular calcification? A systematic review. Nutrients.

[100] de Vries F., Bittner R., Maresz K., Machuron F., Gåserød O., Jeanne J.-F., Schurgers L.J. (2025). Effects of one-year menaquinone-7 supplementation on vascular stiffness and blood pressure in post-menopausal women. Nutrients.

[101] Yu W.Y.-H., Bhutani T., Kornik R. (2017). Warfarin-associated nonuremic calciphylaxis. JAMA Dermatol..

[102] Patrono C., Coller B., FitzGerald G.A., Hirsh J., Roth G. (2004). Platelet-active drugs: The relationships among dose, effectiveness, and side effects: The Seventh ACCP Conference on Antithrombotic and Thrombolytic Therapy. Chest.

[103] Vilahur G., Fuster V. (2025). Interplay between platelets and coagulation: From protective haemostasis to pathological arterial thrombosis. Eur. Heart J..

[104] Davì G., Patrono C. (2007). Platelet activation and atherothrombosis. N. Engl. J. Med..

[105] Grosser N., Schröder H. (2003). Aspirin protects endothelial cells from oxidant damage via the nitric oxide-cGMP pathway. Arterioscler. Thromb. Vasc. Biol..

[106] Li X., Zhang G., Cao X. (2023). The function and regulation of platelet P2Y12 receptor. Cardiovasc. Drugs Ther..

[107] Gao Y., Yu C., Pi S., Mao L., Hu B. (2019). The role of P2Y12 receptor in ischemic stroke of atherosclerotic origin. Cell. Mol. Life Sci..

[108] Didehvar K., Haghshenas M., Yarmohammadi R., Hajikarimloo B., Ghafoury R. (2025). Tissue plasminogen activator as an approved strategy for ischemic stroke: A review of tPA’s structure, mechanism of action and the novel targeting methods. J. Mol. Neurosci..

[109] Zhu J., Wan Y., Xu H., Wu Y., Hu B., Jin H. (2019). The role of endogenous tissue-type plasminogen activator in neuronal survival after ischemic stroke: Friend or foe?. Cell. Mol. Life Sci..

[110] Zhao M., Wang J., Liu G., Li S., Ding Y., Ji X., Zhao W. (2024). Multi-target and multi-phase adjunctive cerebral protection for acute ischemic stroke in the reperfusion era. Biomolecules.

[111] Yang Y., Guo D., Liu Y., Li Y. (2024). Advances in neuroprotective therapy for acute ischemic stroke. Explor. Neuroprot. Ther..

[112] Kennedy J.W. (1999). Thrombolytic therapy in acute myocardial infarction. J. Am. Coll. Cardiol..

[113] Patriarcheas V., Pikoulas A., Kostis M., Charpidou A., Dimakakos E. (2020). Heparin-induced thrombocytopenia: Pathophysiology, diagnosis and management. Cureus.

[114] Comp P.C., Elrod J.P., Karzenski S. (1990). Warfarin-induced skin necrosis. Semin. Thromb. Hemost..

[115] Osman R.M., Suliman A., Mohamedosman R., Tahameed T., Mohamed E. (2025). Warfarin-induced skin necrosis: A narrative review of clinical features, risk factors, and treatment strategies. Ann. Med. Surg..

[116] Grip L., Blombäck M., Schulman S. (1991). Hypercoagulable state and thromboembolism following warfarin withdrawal in post-myocardial-infarction patients. Eur. Heart J..

[117] Junqueira D.R., Zorzela L.M., Perini E. (2017). Unfractionated heparin versus low molecular weight heparins for avoiding heparin-induced thrombocytopenia in postoperative patients. Cochrane Database Syst. Rev..

[118] Chen L.-Y., Khan N., Lindenbauer A., Nguyen T.-H. (2022). When will fondaparinux induce thrombocytopenia?. Bioconjug Chem..

[119] Krauel K., Fürll B., Warkentin T.E., Weitschies W., Kohlmann T., Sheppard J.I., Greinacher A. (2008). Heparin-induced thrombocytopenia—Therapeutic concentrations of danaparoid, unlike fondaparinux and direct thrombin inhibitors, inhibit formation of platelet factor 4-heparin complexes. J. Thromb. Haemost..

[120] Smythe M.A., Priziola J., Dobesh P.P., Wirth D., Cuker A., Wittkowsky A.K. (2016). Guidance for the practical management of the heparin anticoagulants in the treatment of venous thromboembolism. J. Thromb. Thrombolysis.

[121] Wigle P., Hein B., Bloomfield H.E., Tubb M., Doherty M. (2013). Updated guidelines on outpatient anticoagulation. Am. Fam. Physician.

[122] Pollack C.V., Reilly P.A., Eikelboom J., Glund S., Verhamme P., Bernstein R.A., Dubiel R., Huisman M.V., Hylek E.M., Kamphuisen P.W. (2015). Idarucizumab for dabigatran reversal. N. Engl. J. Med..

[123] Pollack C.V., Reilly P.A., van Ryn J., Eikelboom J.W., Glund S., Bernstein R.A., Dubiel R., Huisman M.V., Hylek E.M., Kam C.-W. (2017). Idarucizumab for dabigatran reversal: Full cohort analysis. N. Engl. J. Med..

[124] Chandiramani A.S., Jenkin I., Botezatu B., Harky A. (2022). Protamine-induced coronary graft thrombosis: A review. J. Cardiothorac. Vasc. Anesth..

[125] Boer C., Meesters M.I., Veerhoek D., Vonk A.B.A. (2018). Anticoagulant and side-effects of protamine in cardiac surgery: A narrative review. Br. J. Anaesth..

[126] Alrayes H., Alsaadi A., Alkhatib A., Patel D.A., Alqarqaz M., Frisoli T., Fuller B., Khandelwal A., Koenig G., O’Neill B.P. (2025). Safety and complications associated with the use of protamine in percutaneous coronary intervention. J. Invasive Cardiol..

[127] Sharifi-Rad J., Sharopov F., Ezzat S.M., Zam W., Ademiluyi A.O., Oyeniran O.H., Adetunji C.O., Roli O.I., Živković J., Martorell M. (2023). An updated review on glycoprotein IIb/IIIa inhibitors as antiplatelet agents: Basic and clinical perspectives. High Blood Press. Cardiovasc. Prev..

[128] Hasan N., Jauregui W., Zubair M., Pushparajan V.K., Carson B.J., Attaluri D.M., Dixon D., Jaisinghani A., Chuecos A., Ravichandran D. (2023). Adverse drug effect profiles of Gp2b/3a inhibitors: A comparative review of the last two decades. Cureus.

[129] Guo Y.-L., Li J.-J., Yuan J.-Q., Qin X.-W., Zheng X., Mu C.-W., Hua Y.-H. (2010). Profound thrombocytopenia induced by clopidogrel with a prior history of long-term safe administration. World J. Cardiol..

[130] Frydrych M., Janeczek M., Małyszek A., Nelke K., Dobrzyński M., Lukaszewski M. (2024). Prothrombotic rebound after discontinuation of direct oral anticoagulants therapy: A systematic review. J. Clin. Med..

[131] Burger W., Chemnitius J.-M., Kneissl G.D., Rücker G. (2005). Low-dose aspirin for secondary cardiovascular prevention—Cardiovascular risks after its perioperative withdrawal versus bleeding risks with its continuation—Review and meta-analysis. J. Intern. Med..

[132] Verstraete M. (2000). Third-generation thrombolytic drugs. Am. J. Med..

[133] Collen D., Lijnen H.R. (1991). Basic and clinical aspects of fibrinolysis and thrombolysis. Blood.

[134] Gold H.K., Leinbach R.C., Garabedian H.D., Yasuda T., Johns J.A., Grossbard E.B., Palacios I., Collen D. (1986). Acute coronary reocclusion after thrombolysis with recombinant human tissue-type plasminogen activator: Prevention by a maintenance infusion. Circulation.

[135] Hoffmeister H.M., Szabo S., Helber U., Seipel L. (2001). The thrombolytic paradox. Thromb. Res..

[136] Napolitano F., Montuori N. (2022). Role of plasminogen activation system in platelet pathophysiology: Emerging concepts for translational applications. Int. J. Mol. Sci..

[137] Taylor F.B., Toh C.H., Hoots W.K., Wada H., Levi M. (2001). Scientific Subcommittee on Disseminated Intravascular Coagulation (DIC) of the International Society on Thrombosis and Haemostasis (ISTH). Towards definition, clinical and laboratory criteria, and a scoring system for disseminated intravascular coagulation. Thromb. Haemost..

[138] Totoki T., Koami H., Makino Y., Wada T., Ito T., Yamakawa K., Iba T. (2024). Heparin therapy in sepsis and sepsis-associated disseminated intravascular coagulation: A systematic review and meta-analysis. Thromb. J..

[139] Rock G.A., Shumak K.H., Buskard N.A., Blanchette V.S., Kelton J.G., Nair R.C., Spasoff R.A. (1991). Canadian Apheresis Study Group. Comparison of plasma exchange with plasma infusion in the treatment of thrombotic thrombocytopenic purpura. N. Engl. J. Med..

[140] Legendre C.M., Licht C., Muus P., Greenbaum L.A., Babu S., Bedrosian C., Bingham C., Cohen D.J., Delmas Y., Douglas K. (2013). Terminal complement inhibitor eculizumab in atypical hemolytic-uremic syndrome. N. Engl. J. Med..

[141] Licht C., Greenbaum L.A., Muus P., Babu S., Bedrosian C.L., Cohen D.J., Delmas Y., Douglas K., Furman R.R., Gaber O.A. (2015). Efficacy and safety of eculizumab in atypical hemolytic uremic syndrome from 2-year extensions of phase 2 studies. Kidney Int..

[142] Drmic D., Samara M., Vidovic T., Malekinusic D., Antunovic M., Vrdoljak B., Ruzman J., Milkovic Perisa M., Horvat Pavlov K., Jeyakumar J. (2018). Counteraction of perforated cecum lesions in rats: Effects of pentadecapeptide BPC 157, L-NAME and L-arginine. World J. Gastroenterol..

[143] Singer A.J., Clark R.A.F. (1999). Cutaneous wound healing. N. Engl. J. Med..

[144] Ruggeri Z.M. (2000). Old concepts and new developments in the study of platelet aggregation. J. Clin. Investig..

[145] Seiwerth S., Milavic M., Vukojevic J., Gojkovic S., Krezic I., Vuletic L.B., Pavlov K.H., Petrovic A., Sikiric S., Vranes H. (2021). Stable gastric pentadecapeptide BPC 157 and wound healing. Front. Pharmacol..

[146] Sikiric P., Seiwerth S., Grabarevic Z., Petek M., Rucman R., Turkovic B., Rotkvic I., Jagic V., Duvnjak M., Mise S. (1994). The beneficial effect of BPC 157, a 15 amino acid peptide BPC fragment, on gastric and duodenal lesions induced by restraint stress, cysteamine and 96% ethanol in rats: A comparative study with H2 receptor antagonists, dopamine promotors and gut peptides. Life Sci..

[147] Wang Q., Liu C., An J., Liu J., Wang Y., Cai Y. (2025). Mechanisms of microbial infection and wound healing in diabetic foot ulcer: Pathogenicity in the inflammatory-proliferative phase, chronicity, and treatment strategies. Front. Endocrinol..

[148] Seveljević-Jaran D., Cuzić S., Dominis-Kramarić M., Glojnarić I., Ivetić V., Radosević S., Parnham M.J. (2006). Accelerated healing of excisional skin wounds by PL 14736 in alloxan-hyperglycemic rats. Ski. Pharmacol. Physiol..

[149] Mikus D., Sikiric P., Seiwerth S., Petricevic A., Aralica G., Druzijancic N., Rucman R., Petek M., Pigac B., Perovic D. (2001). Pentadecapeptide BPC 157 cream improves burn-wound healing and attenuates burn-gastric lesions in mice. Burns.

[150] Sikiric P., Seiwerth S., Mise S., Staresinic M., Bedekovic V., Zarkovic N., Borovic S., Gjurasin M., Boban-Blagaic A., Batelja L. (2003). Corticosteroid-impairment of healing and gastric pentadecapeptide BPC-157 creams in burned mice. Burns.

[151] Bilic M., Bumber Z., Blagaic A.B., Batelja L., Seiwerth S., Sikiric P. (2005). The stable gastric pentadecapeptide BPC 157, given locally, improves CO_2_ laser healing in mice. Burns.

[152] Seiwerth S., Sikiric P., Grabarevic Z., Zoricic I., Hanzevacki M., Ljubanovic D., Coric V., Konjevoda P., Petek M., Rucman R. (1997). BPC 157’s effect on healing. J. Physiol. Paris.

[153] Krivic A., Anic T., Seiwerth S., Huljev D., Sikiric P. (2006). Achilles detachment in rat and stable gastric pentadecapeptide BPC 157: Promoted tendon-to-bone healing and opposed corticosteroid aggravation. J. Orthop. Res..

[154] Krivic A., Majerovic M., Jelic I., Seiwerth S., Sikiric P. (2008). Modulation of early functional recovery of Achilles tendon to bone unit after transection by BPC 157 and methylprednisolone. Inflamm. Res..

[155] Staresinic M., Sebecic B., Patrlj L., Jadrijevic S., Suknaic S., Perovic D., Aralica G., Zarkovic N., Borovic S., Srdjak M. (2003). Gastric pentadecapeptide BPC 157 accelerates healing of transected rat Achilles tendon and in vitro stimulates tendocytes growth. J. Orthop. Res..

[156] Brcic L., Brcic I., Staresinic M., Novinscak T., Sikiric P., Seiwerth S. (2009). Modulatory effect of gastric pentadecapeptide BPC 157 on angiogenesis in muscle and tendon healing. J. Physiol. Pharmacol..

[157] Sikiric P., Buljan M., Vnuk D., Krstonijevic Z., Sever M., Lojo N., Drmic D., Zenko Sever A., Baric M., Starcevic N. (2014). Effect of pentadecapeptide BPC 157 on rotator cuff tear injury in rat. FASEB J..

[158] Jukic I., Kokic N., Sikiric P., Mise S., Seiwerth S., Anic T., Jukic J. (2003). Gastric pentadecapeptide BPC 157 heals myofibrotic contracture induced by multiple blunt leg injury in the rat. Dig. Dis. Sci..

[159] Cerovecki T., Bojanic I., Brcic L., Radic B., Vukoja I., Seiwerth S., Sikiric P. (2010). Pentadecapeptide BPC 157 (PL 14736) improves ligament healing in the rat. J. Orthop. Res..

[160] Staresinic M., Petrovic I., Novinscak T., Jukic I., Pevec D., Suknaic S., Kokic N., Brcic L., Seiwerth S., Sikiric P. (2006). Effective therapy of transected quadriceps muscle in rat: Gastric pentadecapeptide BPC 157. J. Orthop. Res..

[161] Novinscak T., Brcic L., Staresinic M., Jukic I., Radic B., Pevec D., Mise S., Tomasovic S., Brcic I., Banic T. (2008). Gastric pentadecapeptide BPC 157 as an effective therapy for muscle crush injury in the rat. Surg. Today.

[162] Pevec D., Novinscak T., Brcic L., Sipos K., Jukic I., Staresinic M., Mise S., Brcic I., Kolenc D., Klicek R. (2010). Impact of pentadecapeptide BPC 157 on muscle healing impaired by systemic corticosteroid application. Med. Sci. Monit..

[163] Mihovil I., Radic B., Brcic L., Brcic I., Vukoja I., Ilic S., Boban Blagaic A., Seiwerth S., Sikiric P. (2009). Beneficial effect of pentadecapeptide BPC 157 on denervated muscle in rats. J. Physiol. Pharmacol..

[164] Japjec M., Horvat Pavlov K., Petrovic A., Staresinic M., Sebecic B., Buljan M., Vranes H., Giljanovic A., Drmic D., Japjec M. (2021). Stable gastric pentadecapeptide BPC 157 as a therapy for the disable myotendinous junctions in rats. Biomedicines.

[165] Matek D., Matek I., Staresinic E., Japjec M., Bojanic I., Boban Blagaic A., Beketic Oreskovic L., Oreskovic I., Ziger T., Novinscak T. (2025). Stable gastric pentadecapeptide BPC 157 as therapy after surgical detachment of the quadriceps muscle from its attachments for muscle-to-bone reattachment in rats. Pharmaceutics.

[166] Masnec S., Kokot A., Zlatar M., Kalauz M., Kunjko K., Radic B., Klicek R., Drmic D., Lazic R., Brcic L. (2015). Perforating corneal injury in rat and pentadecapeptide BPC 157. Exp. Eye Res..

[167] Bajramagic S., Sever M., Rasic F., Staresinic M., Skrtic A., Beketic Oreskovic L., Oreskovic I., Strbe S., Loga Zec S., Hrabar J. (2024). Stable gastric pentadecapeptide BPC 157 and intestinal anastomoses therapy in rats—A review. Pharmaceuticals.

[168] Sikiric P., Drmic D., Sever M., Klicek R., Blagaic A.B., Tvrdeic A., Kralj T., Kovac K.K., Vukojevic J., Siroglavic M. (2020). Fistulas healing: Stable gastric pentadecapeptide BPC 157 therapy. Curr. Pharm. Des..

[169] Skorjanec S., Dolovski Z., Kocman I., Brcic L., Blagaic Boban A., Batelja L., Coric M., Sever M., Klicek R., Berkopic L. (2009). Stable gastric pentadecapeptide BPC 157 heals rat colovesical fistula: Therapy for unhealed gastrocutaneous fistulas in rats as a model for analogous healing of persistent skin wounds and persistent gastric ulcers. Dig. Dis. Sci..

[170] Klicek R., Sever M., Radic B., Drmic D., Kocman I., Zoricic I., Vuksic T., Ivica M., Barisic I., Ilic S. (2008). Pentadecapeptide BPC 157, in clinical trials as a therapy for inflammatory bowel disease (PL14736), is effective in the healing of colocutaneous fistulas in rats: Role of the nitric oxide-system. J. Pharmacol. Sci..

[171] Skorjanec S., Kokot A., Drmic D., Radic B., Sever M., Klicek R., Kolenc D., Zenko A., Lovric Bencic M., Belosic Halle Z. (2015). Duodenocutaneous fistula in rats as a model for “wound healing-therapy”: The effect of pentadecapeptide BPC 157, L-nitro-arginine methyl ester and L-arginine. J. Physiol. Pharmacol..

[172] Cesarec V., Becejac T., Misic M., Djakovic Z., Olujic D., Drmic D., Brcic L., Rokotov D.S., Seiwerth S., Sikiric P. (2013). Pentadecapeptide BPC 157 and esophagocutaneous fistula healing therapy. Eur. J. Pharmacol..

[173] Madzarac G., Becejac T., Penovic T., Drazenovic D., Kralj L., Popović Dolić M., Sikiric S., Beketic Oreskovic L., Oreskovic I., Strbe S. (2026). Tracheocutaneous fistula resolved by pentadecapeptide BPC 157 therapy through the NO-system—Triple NO-agent approach in rats. Pharmaceuticals.

[174] Vukusic D., Zenko Sever A., Sever M., Drmic D., Milavic M., Sikiric S., Rasic D., Krezic I., Gojkovic S., Prtoric A. (2024). Duodenocolic fistula healing by pentadecapeptide BPC 157 in rats: A cytoprotection viewpoint. J. Physiol. Pharmacol..

[175] Rasic D., Zenko Sever A., Rasic F., Strbe S., Rasic Z., Djuzel A., Duplancic B., Boban Blagaic A., Skrtic A., Seiwerth S. (2021). Stable gastric pentadecapeptide BPC 157 heals established vesicovaginal fistula and counteracts stone formation in rats. Biomedicines.

[176] Grgic T., Grgic D., Drmic D., Sever A.Z., Petrovic I., Sucic M., Kokot A., Klicek R., Sever M., Seiwerth S. (2016). Assessment of colovesical fistula healing in rats by pentadecapeptide BPC 157. Eur. J. Pharmacol..

[177] Baric M., Sever A.Z., Vuletic L.B., Rasic Z., Sever M., Drmic D., Pavelic-Turudic T., Sucic M., Vrcic H., Seiwerth S. (2016). Stable gastric pentadecapeptide BPC 157 heals rectovaginal fistula in rats. Life Sci..

[178] Rahman O.F., Lee S.J., Seeds W.A. (2026). Therapeutic peptides in orthopaedics: Applications, challenges, and future directions. J. Am. Acad. Orthop. Surg. Glob. Res. Rev..

[179] Mayfield C.K., Bolia I.K., Feingold C.L., Lin E.H., Liu J.N., Rick Hatch G.F., Gamradt S.C., Weber A.E. (2026). Injectable peptide therapy: A primer for orthopaedic and sports medicine physicians. Am. J. Sports Med..

[180] DeFoor M.T., Dekker T.J. (2025). Injectable therapeutic peptides—An adjunct to regenerative medicine and sports performance?. Arthroscopy.

[181] Giandonato J.A., Tringali V. (2025). Pentadecapeptide BPC 157: Panacea or overhyped peptide?. Eur. J. Phys. Educ. Sport Sci..

[182] McGuire F.P., Martinez R., Lenz A., Skinner L., Cushman D.M. (2025). Regeneration or risk? A narrative review of BPC 157 for musculoskeletal healing. Curr. Rev. Musculoskelet. Med..

[183] Dekker T.J. (2025). Editorial commentary: Testosterone, growth hormone, and vitamin D supplementation is not routinely indicated for orthopaedic surgery patients. Arthroscopy.

[184] Vasireddi N., Hahamyan H., Salata M.J., Karns M., Calcei J.G., Voos J.E., Apostolakos J.M. (2025). Emerging use of BPC 157 in orthopaedic sports medicine: A systematic review. HSS J..

[185] Whitehouse M. (2025). Concerning BPC-157, a natural pentadecapeptide, that acts as a cytoprotectant and is believed to protect the gastro-intestinal tract (GIT). Inflammopharmacology.

[186] DiStefano M.J., Dardouri M., Moore G.D., Saseen J.J., Nair K.V. (2025). Compounded glucagon-like peptide-1 receptor agonists for weight loss: The direct-to-consumer market in Colorado. J. Pharm. Policy Pract..

[187] Chan M.K.S., Wong M.B.F., Chernykh V., Iemeliyanova M., Alvin G., Nishkumai O., Lakey J.R.T., Klokol D. (2025). Emerging anabolic and regenerative peptides in athletic body re-composition and bodybuilding: Mechanisms of action, dosing strategies, and evidence review. J. Stem Cell Res..

[188] Kim G., Fang W., Chung J., Dunn E., Lin R., Mo K., Bascharon R., McGee R. (2025). Application of peptide therapy for ligaments and tendons: A narrative review. J. Orthop. Rep..

[189] Cushman C.J., Ibrahim A.F., Smith A.D., Hernandez E.J., MacKay B., Zumwalt M. (2024). Local and systemic peptide therapies for soft tissue regeneration: A narrative review. Yale J. Biol. Med..

[190] Gwyer D., Wragg N.M., Wilson S.L. (2019). Gastric pentadecapeptide body protection compound BPC 157 and its role in accelerating musculoskeletal soft tissue healing. Cell Tissue Res..

[191] Kim A.T., Sneistrup C., Berg T.M. (2017). Prostaglandin E1 increases microcirculation in random pattern flaps on rats measured with laser Doppler perfusion imaging. Plast. Reconstr. Surg. Glob. Open.

[192] Takahashi S., Tagami Y., Maie O. (1994). Clinical effects of G-511 ointment (prostaglandin E1 ointment) on the treatment of chronic skin ulcers. Rinshouiyaku.

[193] Duthois S., Cailleux N., Benosman B., Lévesque H. (2003). Tolerance of iloprost and results of treatment of chronic severe lower limb ischaemia in diabetic patients: A retrospective study of 64 consecutive cases. Diabetes Metab..

[194] Zhang J.Z., Maruyama K., Iwatsuki K., Ono I., Kaneko F. (1994). Effect of prostaglandin E_1_ on human keratinocytes and dermal fibroblasts: A possible mechanism for the healing of skin ulcers. Exp. Dermatol..

[195] White E.S., Atrasz R.G., Dickie E.G., Aronoff D.M., Stambolic V., Mak T.W., Moore B.B., Peters-Golden M. (2005). Prostaglandin E_2_ inhibits fibroblast migration by E-prostanoid 2 receptor-mediated increase in PTEN activity. Am. J. Respir. Cell Mol. Biol..

[196] Zahedipour F., Butler A.E., Eid A.H., Sahebkar A. (2022). Pleiotropic properties of statins via angiogenesis modulation in cardiovascular disease. Drug Discov. Today.

[197] Torgal S.S., Hiremath S.V., Majagi S.I., Gouripur V.V., Patil P.A., Hogade A.P. (2010). Evaluation of wound healing activity of angiotensin converting enzyme inhibitors in Wistar rats. Recent Res. Sci. Technol..

[198] Fang Q.-Q., Wang X.-F., Zhao W.-Y., Ding S.-L., Shi B.-H., Xia Y., Yang H., Wu L.-H., Li C.-Y., Tan W.-Q. (2018). Angiotensin-converting enzyme inhibitor reduces scar formation by inhibiting both canonical and noncanonical TGF-β1 pathways. Sci. Rep..

[199] Chen J.D., Liu M., Chen X.H., Yang Z.J. (2015). Effect of angiotensin receptor blockers on flow-mediated vasodilation: A meta-analysis of randomized controlled trials. Cardiology.

[200] Jia S., Wang X., Wang G., Wang X. (2024). Mechanism and application of β-adrenoceptor blockers in soft tissue wound healing. Med. Res. Rev..

[201] Romana-Souza B., Porto L.C., Monte-Alto-Costa A. (2010). Cutaneous wound healing of chronically stressed mice is improved through catecholamines blockade. Exp. Dermatol..

[202] Ali A., Herndon D.N., Mamachen A., Hasan S., Andersen C.R., Grogans R.-J., Brewer J.L., Lee J.O., Heffernan J., Suman O.E. (2015). Propranolol attenuates hemorrhage and accelerates wound healing in severely burned adults. Crit. Care.

[203] Assis de Brito T.L., Monte-Alto-Costa A., Romana-Souza B. (2014). Propranolol impairs the closure of pressure ulcers in mice. Life Sci..

[204] Raut S.B., Nerlekar S.R., Pawar S., Patil A.N. (2012). An evaluation of the effects of nonselective and cardioselective β-blockers on wound healing in Sprague Dawley rats. Indian J. Pharmacol..

[205] Zolfagharnezhad H., Khalili H., Mohammadi M., Niknam S., Vatanara A. (2021). Topical nifedipine for the treatment of pressure ulcer: A randomized, placebo-controlled clinical trial. Am. J. Ther..

[206] Teimouri A., Yeung P., Agu R.U. (2020). Drug-release assessment of compounded topical nifedipine and diltiazem in commonly used bases for wound healing. Int. J. Pharm. Compd..

[207] Brasileiro A.C.L., de Oliveira D.C., da Silva P.B., Rocha J.K.S.L. (2020). Impact of topical nifedipine on wound healing in animal model (pig). J. Vasc. Bras..

[208] Mojiri-Forushani H. (2018). The role of calcium channel blockers in wound healing. Iran. J. Basic Med. Sci..

[209] Bagheri M., Jahromi B.M., Mirkhani H., Solhjou Z., Noorafshan A., Zamani A., Amirghofran Z. (2011). Azelnidipine, a new calcium channel blocker, promotes skin wound healing in diabetic rats. J. Surg. Res..

[210] Bhasker H., Udupa S.L., Udupa A.L. (2004). Effect of nifedipine and amlodipine on wound healing in rats. Indian J. Physiol. Pharmacol..

[211] Hemmati A.A., Forushani H.M., Asgari H.M. (2014). Wound healing potential of topical amlodipine in full thickness wound of rabbit. Jundishapur J. Nat. Pharm. Prod..

[212] Schwentker A., Billiar T.R. (2003). Nitric oxide and wound repair. Surg. Clin. N. Am..

[213] Luo J.D., Chen A.F. (2005). Nitric oxide: A newly discovered function on wound healing. Acta Pharmacol. Sin..

[214] Witte M.B., Kiyama T., Barbul A. (2002). Nitric oxide enhances experimental wound healing in diabetes. Br. J. Surg..

[215] Shabani M., Pulfer S.K., Bulgrin J.P., Smith D.J. (1996). Enhancement of wound repair with a topically applied nitric oxide-releasing polymer. Wound Repair. Regen..

[216] Amadeu T.P., Seabra A.B., de Oliveira M.G., Monte-Alto-Costa A. (2008). Nitric oxide donor improves healing if applied on inflammatory and proliferative phase. J. Surg. Res..

[217] Stallmeyer B., Kämpfer H., Kolb N., Pfeilschifter J., Frank S. (1999). The function of nitric oxide in wound repair: Inhibition of inducible nitric oxide-synthase severely impairs wound reepithelialization. J. Investig. Dermatol..

[218] Yamasaki K., Edington H.D.J., McClosky C., Tzeng E., Lizonova A., Kovesdi I., Steed D.L., Billiar T.R. (1998). Reversal of impaired wound repair in iNOS-deficient mice by topical adenoviral-mediated iNOS gene transfer. J. Clin. Investig..

[219] Amadeu T.P., Monte-Alto-Costa A.M. (2006). Nitric oxide synthesis inhibition alters rat cutaneous wound healing. J. Cutan. Pathol..

[220] Norgren L., Alwmark A., Angqvist K.A., Hedberg B., Bergqvist D., Takolander R., Claes G., Lundell A., Holm J., Jivegård L. (1990). A stable prostacyclin analogue (iloprost) in the treatment of ischaemic ulcers of the lower limb: A Scandinavian-Polish placebo-controlled, randomised multicenter study. Eur. J. Vasc. Surg..

[221] Pedersen A.K., FitzGerald G.A. (1984). Dose-related kinetics of aspirin: Presystemic acetylation of platelet cyclooxygenase. N. Engl. J. Med..

[222] Gunji H., Ono I., Tateshita T., Kaneko F. (1996). Clinical effectiveness of an ointment containing prostaglandin E1 for the treatment of burn wounds. Burns.

[223] Kristensen K.E., Gislason G.H., Torp-Pedersen C., Rasmussen H.B., Hansen P.R. (2013). Risk of hemorrhagic events in patients co-treated with clopidogrel and angiotensin-converting enzyme inhibitors after myocardial infarction. Eur. Heart J..

[224] Chen R., Suchard M.A., Krumholz H.M., Schuemie M.J., Shea S., Duke J., Pratt N., Reich C.G., Madigan D., You S.C. (2021). Comparative first-line effectiveness and safety of ACE (angiotensin-converting enzyme) inhibitors and angiotensin receptor blockers: A multinational cohort study. Hypertension.

[225] (2000). The Heart Outcomes Prevention Evaluation Study Investigators. Effects of an angiotensin-converting–enzyme inhibitor, ramipril, on cardiovascular events in high-risk patients. N. Engl. J. Med..

[226] Pfeffer M.A., McMurray J.J., Velazquez E.J., Rouleau J.-L., Køber L., Maggioni A.P., Solomon S.D., Swedberg K., Van de Werf F., White H. (2003). Valsartan, captopril, or both in myocardial infarction complicated by heart failure, left ventricular dysfunction, or both. N. Engl. J. Med..

[227] Chae Y.K., Khemasuwan D., Dimou A., Neagu S., Chebrolu L., Gupta S., Carpio A., Kim J., Yun J.H., Smyrlis A. (2014). Inhibition of renin angiotensin axis may be associated with reduced risk of developing venous thromboembolism in patients with atherosclerotic disease. PLoS ONE.

[228] Hannedouche T., Godin M., Fries D., Fillastre J.P. (1991). Acute renal thrombosis induced by angiotensin-converting enzyme inhibitors in patients with renovascular hypertension. Nephron.

[229] Dussol B., Nicolino F., Brunet P., Leonetti F., Siles S., Berland Y. (1994). Acute transplant artery thrombosis induced by angiotensin-converting inhibitor in a patient with renovascular hypertension. Nephron.

[230] Kerbert A.J.C., Chiang F.W.T., van der Werf M., Stijnen T., Slingerland H., Verspaget H.W., van Hoek B., Coenraad M.J. (2017). Hemodynamic response to primary prophylactic therapy with nonselective β-blockers is related to a reduction of first variceal bleeding risk in liver cirrhosis: A meta-analysis. Eur. J. Gastroenterol. Hepatol..

[231] Xu S., Li Z., Yang T., Li L., Song X., Hao Y., Smith S.C., Fonarow G.C., Morgan L., Liu J. (2023). Association between early oral β-blocker therapy and risk for in-hospital major bleeding after percutaneous coronary intervention for acute coronary syndrome: Findings from CCC-ACS project. Eur. Heart J. Qual. Care Clin. Outcomes.

[232] Asadbeygi A., Lee S., Kovalchin J., Hatoum H. (2023). Effect of beta blockers on the hemodynamics and thrombotic risk of coronary artery aneurysms in Kawasaki disease. J. Cardiovasc. Transl. Res..

[233] He Y., Chan E.W., Leung W.K., Anand S., Wong I.C.K. (2015). Systematic review with meta-analysis: The association between the use of calcium channel blockers and gastrointestinal bleeding. Aliment. Pharmacol. Ther..

[234] Sakakibara F., Ueda S., Uchida K., Kinjo N., Arai H., Nezu M., Morimoto T. (2022). Association between dihydropyridine calcium channel blockers and ischemic strokes in patients with nonvalvular atrial fibrillation. Hypertens. Res..

[235] Uchida K., Ueda S., Sakakibara F., Kinjo N., Nezu M., Arai H., Morimoto T. (2023). Statins reduce bleeding risk in patients taking oral anticoagulants for nonvalvular atrial fibrillation: A retrospective registry study. Am. J. Cardiovasc. Drugs.

[236] Agarwal V., Phung O.J., Tongbram V., Bhardwaj A., Coleman C.I. (2010). Statin use and the prevention of venous thromboembolism: A meta-analysis. Int. J. Clin. Pract..

[237] Radomski M.W., Palmer R.M.J., Moncada S. (1990). An L-arginine/nitric oxide pathway present in human platelets regulates aggregation. Proc. Natl. Acad. Sci. USA.

[238] Freedman J.E., Loscalzo J. (2003). Nitric oxide and its relationship to thrombotic disorders. J. Thromb. Haemost..

[239] Wu J., Ye J., Zhu J., Xiao Z., He C., Shi H., Wang Y., Lin C., Zhang H., Zhao Y. (2016). Heparin-based coacervate of FGF2 improves dermal regeneration by asserting a synergistic role with cell proliferation and endogenous facilitated VEGF for cutaneous wound healing. Biomacromolecules.

[240] Galvan L. (1996). Effects of heparin on wound healing. J. Wound Ostomy Cont. Nurs..

[241] Kohyama K., Kato H., Okada H., Ishihara T., Yasue Y., Kamidani R., Suzuki K., Miyake T., Okuda H., Shibata H. (2024). Concomitant heparin use promotes skin graft donor site healing by basic fibroblast growth factor: A pilot prospective randomized controlled study. Contemp. Clin. Trials Commun..

[242] Matzsch T., Bergqvist D., Blomquist P., Jiborn H. (1987). Influence of standard heparin or low molecular weight heparin on healing of abdominal wounds and colonic anastomoses in rats. Acta Chir. Scand..

[243] Civelek A., Ak K., Kurtkaya O., Tekeli A., Isbir S., Nargileci E., Arsan S., Sav A. (2007). Effect of a low molecular weight heparin molecule, dalteparin, on cellular apoptosis and inflammatory process in an incisional wound-healing model. Surg. Today.

[244] Ozdamar Fuad O., Yildirim A.O., Gulcek M., Unal V.S., Karakuyu A., Ozlu K., Ucaner A. (2009). The effect of prophylactic dose of a low molecular weight heparin on skin wound healing of rats. Acta Cir. Bras..

[245] Seebauer C., Hanafieh F., Wolff J., Metelmann H.-R., Vollmer M. (2025). Influence of anticoagulant concomitant medication on wound healing: Analysis of a multicentre cohort of 212 patients with a uniform wound model. Br. J. Clin. Pharmacol..

[246] Lindner T., Cockbain A.J., El Masry M.A., Katonis P., Tsiridis E., Schizas C., Tsiridis E. (2008). The effect of anticoagulant pharmacotherapy on fracture healing. Expert. Opin. Pharmacother..

[247] Ad-El D.D., Meirovitz A., Weinberg A., Kogan L., Arieli D., Neuman A., Linton D. (2000). Warfarin skin necrosis: Local and systemic factors. Br. J. Plast. Surg..

[248] Ma L., Elliott S.N., Cirino G., Buret A., Ignarro L.J., Wallace J.L. (2001). Platelets modulate gastric ulcer healing: Role of endostatin and vascular endothelial growth factor release. Proc. Natl. Acad. Sci. USA.

[249] dos Santos J.S., Monte-Alto-Costa A. (2013). Female, but not male, mice show delayed cutaneous wound healing following aspirin administration. Clin. Exp. Pharmacol. Physiol..

[250] Takahashi K., Nosaka T., Murata Y., Sugata R., Akazawa Y., Tanaka T., Naito T., Matsuda H., Ohani M., Suto H. (2025). Influence of antiplatelet drugs on gastric ulcer healing after endoscopic submucosal dissection in patients with early gastric cancer. DEN Open.

[251] Chan J.C.Y., Duszczyszyn D.A., Castellino F.J., Ploplis V.A. (2001). Accelerated skin wound healing in plasminogen activator inhibitor-1-deficient mice. Am. J. Pathol..

[252] Lund L.R., Rømer J., Bugge T.H., Nielsen B.S., Frandsen T.L., Degen J.L., Stephens R.W., Danø K. (1999). Functional overlap between two classes of matrix-degrading proteases in wound healing. EMBO J..

[253] Lund L.R., Green K.A., Stoop A.A., Ploug M., Almholt K., Lilla J., Nielsen B.S., Christensen I.J., Craik C.S., Werb Z. (2006). Plasminogen activation independent of uPA and tPA maintains wound healing in gene-deficient mice. EMBO J..

[254] Gailani D., Cheng Q., Ivanov I.S. (2015). Murine models in the evaluation of heparan sulfate-based anticoagulants. Methods Mol. Biol..

[255] Palm M., Mattsson C., Svahn C.M., Weber M. (1990). Bleeding times in rats treated with heparin, heparin fragments of high and low anticoagulant activity and chemically modified heparin fragments of low anticoagulant activity. Thromb. Haemost..

[256] Tuthill D.D., Bayer V., Gallagher A.M., Drohan W.N., MacPhee M.J. (2001). Assessment of topical hemostats in a renal hemorrhage model in heparinized rats. J. Surg. Res..

[257] Foerch C., Arai K., Jin G., Park K.P., Pallast S., van Leyen K., Lo E.H. (2008). Experimental model of warfarin-associated intracerebral hemorrhage. Stroke.

[258] Elg M., Gustafsson D., Carlsson S. (1999). Antithrombotic effects and bleeding time of thrombin inhibitors and warfarin in the rat. Thromb. Res..

[259] López-Belmonte J., Whittle B.J.R., Moncada S. (1993). The actions of nitric oxide donors in the prevention or induction of injury to the rat gastric mucosa. Br. J. Pharmacol..

[260] Whittle B.J.R., Boughton-Smith N.K., Moncada S. (1992). Biosynthesis and role of the endothelium-derived vasodilator, nitric oxide, in the gastric mucosa. Ann. N. Y. Acad. Sci..

[261] Palmer R.M., Ferrige A.G., Moncada S. (1987). Nitric oxide release accounts for the biological activity of endothelium-derived relaxing factor. Nature.

[262] Sikiric P., Barisic I., Udovicic M., Lovrić Benčić M., Balenovic D., Strinic D., Zivanovic Posilovic G., Uzun S., Vranes H., Krezic I. (2026). Conventional antiarrhythmics class I–IV, late INa inhibitors, IKs enhancers, RyR2 stabilizers, gap junction modulators, atrial-selective antiarrhythmics, and stable gastric pentadecapeptide BPC 157 as useful cytoprotective therapy in arrhythmias. Pharmaceuticals.

[263] Blackshear J.L., Odell J.A. (1996). Appendage obliteration to reduce stroke in cardiac surgical patients with atrial fibrillation. Ann. Thorac. Surg..

[264] Lip G.Y.H., Proietti M., Potpara T., Mansour M., Savelieva I., Tse H.F., Goette A., Camm A.J., Blomstrom-Lundqvist C., Gupta D. (2023). Atrial fibrillation and stroke prevention: 25 years of research at EP Europace journal. Europace.

[265] Guazzi M., Arena R. (2009). Endothelial dysfunction and pathophysiological correlates in atrial fibrillation. Heart.

[266] Corban M.T., Toya T., Ahmad A., Lerman L.O., Lee H.-C., Lerman A. (2021). Atrial fibrillation and endothelial dysfunction: A potential link?. Mayo Clin. Proc..

[267] Badimon L., Cubedo J. (2017). Hypercoagulability and atrial fibrillation: A two-way road?. Eur. Heart J..

[268] Balenovic D., Bencic M.L., Udovicic M., Simonji K., Hanzevacki J.S., Barisic I., Kranjcevic S., Prkacin I., Coric V., Brcic L. (2009). Inhibition of methyldigoxin-induced arrhythmias by pentadecapeptide BPC 157: A relation with NO-system. Regul. Pept..

[269] Barisic I., Balenovic D., Klicek R., Radic B., Nikitovic B., Drmic D., Udovicic M., Strinic D., Bardak D., Berkopic L. (2013). Mortal hyperkalemia disturbances in rats are NO-system related: The life saving effect of pentadecapeptide BPC 157. Regul. Pept..

[270] Balenovic D., Barisic I., Prkacin I., Horvat I., Udovicic M., Uzun S., Strinic D., Pevec D., Drmic D., Radic B. (2012). Mortal furosemide-hypokalemia-disturbances in rats NO-system related shorten survival by L-NAME. Therapy benefit with BPC 157 peptide more than with L-arginine. J. Clin. Exp. Cardiolog..

[271] Lozic M., Stambolija V., Krezic I., Dugandzic A., Zivanovic-Posilovic G., Gojkovic S., Kovacevic J., Vrdoljak L., Mirkovic I., Kokot A. (2020). In relation to NO-system, stable pentadecapeptide BPC 157 counteracts lidocaine-induced adverse effects in rats and depolarisation in vitro. Emerg. Med. Int..

[272] Zivanovic-Posilovic G., Balenovic D., Barisic I., Strinic D., Stambolija V., Udovicic M., Uzun S., Drmic D., Vlainic J., Bencic M.L. (2016). Stable gastric pentadecapeptide BPC 157 and bupivacaine. Eur. J. Pharmacol..

[273] Stambolija V., Perleta Stambolija T., Katancic Holjevac J., Murselovic T., Radonic J., Duzel V., Duplancic B., Uzun S., Zivanovic-Posilovic G., Kolenc D. (2016). BPC 157: The counteraction of succinylcholine, hyperkalemia, and arrhythmias. Eur. J. Pharmacol..

[274] Strinić D., Belosić Halle Ž., Luetić K., Nedić A., Petrović I., Sucić M., Živanović Posilović G., Balenović D., Strbe S., Udovičić M. (2017). BPC 157 counteracts QTc prolongation induced by haloperidol, fluphenazine, clozapine, olanzapine, quetiapine, sulpiride, and metoclopramide in rats. Life Sci..

[275] Udovicic M., Sever M., Kavur L., Loncaric K., Barisic I., Balenovic D., Zivanovic Posilovic G., Strinic D., Uzun S., Batelja Vuletic L. (2021). Stable gastric pentadecapeptide BPC 157 therapy for monocrotaline-induced pulmonary hypertension in rats leads to prevention and reversal. Biomedicines.

[276] Medvidovic-Grubisic M., Stambolija V., Kolenc D., Katancic J., Murselovic T., Plestina-Borjan I., Strbe S., Drmic D., Barisic I., Sindic A. (2017). Hypermagnesemia disturbances in rats, NO-related: Pentadecapeptide BPC 157 abrogates, L-NAME and L-arginine worsen. Inflammopharmacology.

[277] Bjerkelund C.J., Orning O.M. (1969). The efficacy of anticoagulant therapy in preventing embolism related to D.C. electrical conversion of atrial fibrillation. Am. J. Cardiol..

[278] Lip G.Y.H., Nieuwlaat R., Pisters R., Lane D.A., Crijns H.J.G.M. (2010). Refining clinical risk stratification for predicting stroke and thromboembolism in atrial fibrillation using a novel risk factor-based approach: The Euro Heart Survey on Atrial Fibrillation. Chest.

[279] Weigner M.J., Caulfield T.A., Danias P.G., Silverman D.I., Manning W.J. (1997). Risk for clinical thromboembolism associated with conversion to sinus rhythm in patients with atrial fibrillation lasting less than 48 hours. Ann. Intern. Med..

[280] Gallagher M.M., Hennessy B.J., Edvardsson N., Hart C.M., Shannon M.S., Obel O.A., Al-Saady N.M., Camm A.J. (2002). Embolic complications of direct current cardioversion of atrial arrhythmias: Association with low intensity of anticoagulation at the time of cardioversion. J. Am. Coll. Cardiol..

[281] Ariëns E.J. (1983). Intrinsic activity: Partial agonists and partial antagonists. J. Cardiovasc. Pharmacol..

[282] Cruickshank J.M. (2007). Are we misunderstanding β-blockers?. Int. J. Cardiol..

[283] Taddei S., Tsabedze N., Tan R.-S. (2024). β-blockers are not all the same: Pharmacologic similarities and differences, potential combinations and clinical implications. Curr. Med. Res. Opin..

[284] Jones K.E., Hayden S.L., Meyer H.R., Sandoz J.L., Arata W.H., Dufrene K., Ballaera C., Lopez Torres Y., Griffin P., Kaye A.M. (2024). The evolving role of calcium channel blockers in hypertension management: Pharmacological and clinical considerations. Curr. Issues Mol. Biol..

[285] January C.T., Wann L.S., Calkins H., Chen L.Y., Cigarroa J.E., Cleveland J.C., Ellinor P.T., Ezekowitz M.D., Field M.E., Furie K.L. (2019). 2019 AHA/ACC/HRS focused update of the 2014 AHA/ACC/HRS guideline for the management of patients with atrial fibrillation: A report of the American College of Cardiology/American Heart Association task force on clinical practice guidelines and the Heart Rhythm Society in collaboration with the Society of Thoracic Surgeons. Circulation.

[286] Dibner-Dunlap M.E., Smith M.L., Kinugawa T., Thames M.D. (1996). Enalaprilat augments arterial and cardiopulmonary baroreflex control of sympathetic nerve activity in patients with heart failure. J. Am. Coll. Cardiol..

[287] Kida T., Sawada K., Kobayashi K., Hori M., Ozaki H., Murata T. (2014). Diverse effects of prostaglandin E2 on vascular contractility. Heart Vessel..

[288] Hristovska A.-M., Rasmussen L.E., Hansen P.B.L., Nielsen S.S., Nüsing R.M., Narumiya S., Vanhoutte P., Skøtt O., Jensen B.L. (2007). Prostaglandin E2 induces vascular relaxation by E-prostanoid 4 receptor-mediated activation of endothelial nitric oxide synthase. Hypertension.

[289] Herring N., Paterson D.J. (2001). Nitric oxide–cGMP pathway facilitates acetylcholine release and bradycardia during vagal nerve stimulation in the guinea-pig in vitro. J. Physiol..

[290] Choate J.K., Danson E.J., Morris J.F., Paterson D.J. (2001). Peripheral vagal control of heart rate is impaired in neuronal NOS knockout mice. Am. J. Physiol. Heart Circ. Physiol..

[291] Pechanova O., Vrankova S., Cebova M. (2009). Chronic L-NAME treatment produces hypertension by different mechanisms in peripheral tissues and brain: Role of central eNOS. Physiol. Res..

[292] Wibawa K., Dewangga R., Nastiti K.S., Syah P.A., Suhendiwijaya S., Ariffudin Y. (2023). Prior statin use and the incidence of in-hospital arrhythmia in acute coronary syndrome: A systematic review and meta-analysis. Indian Heart J..

[293] Wanahita N., Chen J., Bangalore S., Shah K., Rachko M., Coleman C.I., Schweitzer P. (2012). The effect of statin therapy on ventricular tachyarrhythmias: A meta-analysis. Am. J. Ther..

[294] Fiedler L., Hallsson L., Tscharre M., Oebel S., Pfeffer M., Schönbauer R., Tokarska L., Stix L., Haiden A., Kraus J. (2021). Upstream statin therapy and long-term recurrence of atrial fibrillation after cardioversion: A propensity-matched analysis. J. Clin. Med..

[295] Millar P.J., Floras J.S. (2014). Statins and the autonomic nervous system. Clin. Sci..

[296] Pedersen T.R. (2010). Pleiotropic effects of statins: Evidence against benefits beyond LDL-cholesterol lowering. Am. J. Cardiovasc. Drugs.

[297] Welzig C.M., Shin D.-G., Park H.-J., Kim Y.-J., Saul J.P., Galper J.B. (2003). Lipid lowering by pravastatin increases parasympathetic modulation of heart rate: Galpha(i2), a possible molecular marker for parasympathetic responsiveness. Circulation.

[298] Gamulin O., Oroz K., Coric L., Krajacic M., Skrabic M., Dretar V., Strbe S., Talapko J., Juzbasic M., Krezic I. (2022). Fourier transform infrared spectroscopy reveals molecular changes in blood vessels of rats treated with pentadecapeptide BPC 157. Biomedicines.

[299] Smoday I.M., Vukovic V., Oroz K., Vranes H., Kalogjera L., Gamulin O., Vlainic J., Milavic M., Sikiric S., Nikolac Gabaj N. (2026). Fourier transform infrared spectroscopic characterization of aortic wall remodeling by stable gastric pentadecapeptide BPC 157 after unilateral adrenalectomy in rats. Pharmaceuticals.

[300] Mustafa A.K., Gadalla M.M., Snyder S.H. (2009). Signaling by gasotransmitters. Sci. Signal..

[301] Seiwerth S., Rucman R., Turkovic B., Sever M., Klicek R., Radic B., Drmic D., Stupnisek M., Misic M., Vuletic L.B. (2018). BPC 157 and standard angiogenic growth factors: Gastrointestinal tract healing, lessons from tendon, ligament, muscle and bone healing. Curr. Pharm. Des..

[302] Lie S.L., Rognes I.N. (2025). Human and animal models for studying hemorrhagic shock. Scand. J. Trauma. Resusc. Emerg. Med..

[303] Ayyoub S., Orriols R., Oliver E., Tura Ceide O. (2023). Thrombosis models: An overview of common in vivo and in vitro models of thrombosis. Int. J. Mol. Sci..

[304] Badimon L. (1997). Models to study thrombotic disorders. Thromb. Haemost..

[305] Badimon L. (2001). Atherosclerosis and thrombosis: Lessons from animal models. Thromb. Haemost..

[306] Demirtaş H., Özer A., Yıldırım A.K., Dursun A.D., Sezen Ş.C., Arslan M. (2025). Protective effects of BPC 157 on liver, kidney, and lung distant organ damage in rats with experimental lower-extremity ischemia–reperfusion injury. Medicina.

[307] Kalogjera L., Ries M., Baudoin T., Ferencic Z., Trotic R., Pegan B. (1997). Dose-dependent protective effect of BPC 157 on capsaicin-induced rhinitis in rats. Eur. Arch. Otorhinolaryngol..

[308] Veljaca M., Lesch C.A., Pllana R., Sanchez B., Chan K., Guglietta A. (1995). BPC-15 reduces trinitrobenzene sulfonic acid-induced colonic damage in rats. J. Pharmacol. Exp. Ther..

[309] Bódis B., Karádi O., Németh P., Dohoczky C., Kolega M., Mózsik G. (1997). Evidence for direct cellular protective effect of PL-10 substances (synthesized parts of body protection compound, BPC) and their specificity to gastric mucosal cells. Life Sci..

[310] Sandor Z.S., Vincze A., Jadus M.R., Dohoczky C., Erceg D., Brajsa K., Kolega M., Szabo S. (1997). The protective effect of newly isolated peptide PL-10 in the iodoacetamide colitis model in rats. Gastroenterology.

[311] Sandor Z., Vincze A., Szabo S. (1996). The protective effect of a recently isolated gastric peptide in acute and chronic gastric injury. FASEB J..

[312] Jung Y.H., Kim H., Kim H., Kim E., Baik J., Kang H. (2022). The anti-nociceptive effect of BPC 157 on the incisional pain model in rats. J. Dent. Anesth. Pain. Med..

[313] Park S.Y., Choi S.R., Kim J.H., Lee S.C., Jeong J.H., Lee T.Y. (2021). Antinociceptive effect of BPC 157 in the formalin induced pain model. Kosin Med. J..

[314] Miyake T., Kung C.K.H., Goldwasser E. (1977). Purification of human erythropoietin. J. Biol. Chem..

[315] Goldwasser E. (1975). Erythropoietin and the differentiation of red blood cells. Fed. Proc..

[316] Holst J.J. (2007). The physiology of glucagon-like peptide 1. Physiol. Rev..

[317] Drucker D.J. (2006). The biology of incretin hormones. Cell Metab..

[318] Nauck M.A., Bartels E., Ørskov C., Ebert R., Creutzfeldt W. (1993). Additive insulinotropic effects of GIP and GLP-1 in humans. J. Clin. Endocrinol. Metab..

[319] Miyata A., Arimura A., Dahl R.R., Minamino N., Uehara A., Jiang L., Culler M.D., Coy D.H. (1989). Isolation of a novel 38 residue-hypothalamic polypeptide which stimulates adenylate cyclase in pituitary cells. Biochem. Biophys. Res. Commun..

[320] Said S.I. (1991). Vasoactive intestinal polypeptide: Biologic role in health and disease. Trends Endocrinol. Metab..

[321] Harmar A.J., Fahrenkrug J., Gozes I., Laburthe M., May V., Pisegna J.R., Vaudry D., Vaudry H., Waschek J.A., Said S.I. (2012). Pharmacology and functions of receptors for vasoactive intestinal peptide and pituitary adenylate cyclase-activating polypeptide: IUPHAR Review 1. Br. J. Pharmacol..

